# Pyridine C(sp^2^)–H bond functionalization under transition-metal and rare earth metal catalysis

**DOI:** 10.3762/bjoc.19.62

**Published:** 2023-06-12

**Authors:** Haritha Sindhe, Malladi Mounika Reddy, Karthikeyan Rajkumar, Akshay Kamble, Amardeep Singh, Anand Kumar, Satyasheel Sharma

**Affiliations:** 1 Department of Medicinal Chemistry, National Institute of Pharmaceutical Education and Research - Ahmedabad, Gandhinagar, Gujarat, 382355, India; 2 Department of Natural Products, National Institute of Pharmaceutical Education and Research - Ahmedabad, Gandhinagar, Gujarat, 382355, India

**Keywords:** C–H functionalization, heterocycles, pyridine, rare earth metal, transition-metal-catalyzed

## Abstract

Pyridine is a crucial heterocyclic scaffold that is widely found in organic chemistry, medicines, natural products, and functional materials. In spite of the discovery of several methods for the synthesis of functionalized pyridines or their integration into an organic molecule, new methodologies for the direct functionalization of pyridine scaffolds have been developed during the past two decades. In addition, transition-metal-catalyzed C–H functionalization and rare earth metal-catalyzed reactions have flourished over the past two decades in the development of functionalized organic molecules of concern. In this review, we discuss recent achievements in the transition-metal and rare earth metal-catalyzed C–H bond functionalization of pyridine and look into the mechanisms involved.

## Introduction

**Figure 1:**
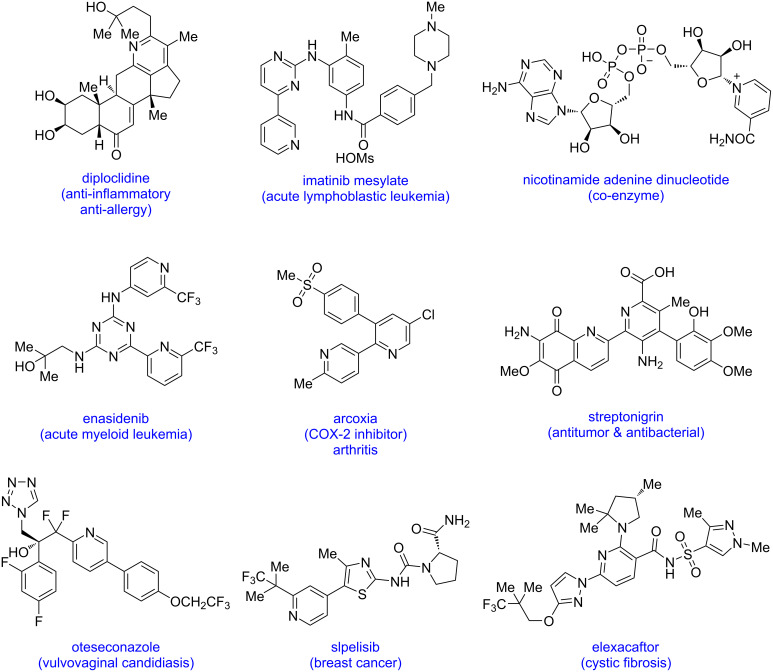
Representative examples of bioactive natural products and FDA-approved drugs containing a pyridine moiety.

Pyridine, one of the most important azaheterocyclic scaffolds, is found in a diverse range of bioactive natural products, pharmaceuticals, and functional materials [1–10]. Due to its different characteristics such as basicity, stability, water solubility, small molecular size, and ability to form hydrogen bonds, pyridine continues to be a suitable moiety in organic synthesis. In addition, it has been observed that pyridine rings serve as bioisostere for aromatic rings, amines, amides, and N-containing heterocycles. Due to the aforementioned qualities, numerous U.S. FDA-approved medications have pyridine scaffolds in their molecules (Figure 1).

**Scheme 1:**
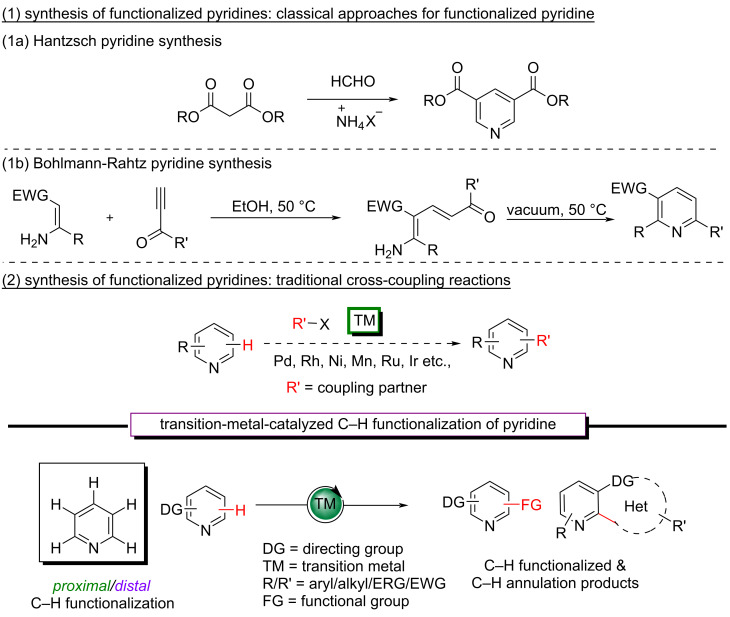
Classical and traditional methods for the synthesis of functionalized pyridines.

In this context, the synthesis of the pyridine motif is always there in trend. Many pyridine syntheses have relied on the condensation of carbonyl compounds and amines for a very long time [11]. The classical methods for the synthesis of functionalized pyridine include the Hantzsch pyridine synthesis and the Bohlmann–Rahtz synthesis (Scheme 1a and b). Furthermore, alternative methodologies are being developed for the synthesis of functionalized pyridines or its integration into an organic molecule [12–20]. Although classical organic synthesis is incredibly effective, it frequently requires the prefunctionalization of substrates and involves stoichiometric waste.

The challenges associated with the functionalization of pyridine are based on the low reactivity of the pyridine ring system for undergoing substitution reactions. This is attributed to the electron-deficient nature of the ring system due to the presence of the sp^2^-hybridized nitrogen atom. In addition, the lone pair electrons of the nitrogen atom interact with Lewis acids instead of the π-electrons of the ring system thus resulting to its reduced reactivity for electrophilic aromatic substitution reactions, such as a Friedel–Crafts reaction [21–23]. Hence, it is challenging to functionalize a C–H bond in pyridine with traditional chemical transformations. On the other hand, intriguing developments have been made for the functionalization of inert C–H bonds in organic synthesis during the past two decades. In this regard, the transition-metal-catalyzed C–H functionalization has made its way towards the synthesis and functionalization of various complex organic molecules [24–31]. In addition, rare earth metal-catalyzed C–H functionalization reactions have been known for a few decades, however, they received growing interest only recently [32–34]. Thus, diversely functionalized pyridines have been synthesized via C–H activation under transition-metal and rare earth metal catalysis, including C–H alkylation, alkenylation, arylation, heteroarylation, borylation, etc. Recently, metal-free approaches have also been developed for the C–H functionalization of N-heterocycles [35–39]. However, due to the vastness of reports on C–H functionalizations of N-heterocycles, in this review we have summarized recent progress (from year 2010 to 2023) in the C–H functionalization of the pyridine ring only. Herein, we discuss transition-metal as well as rare earth metal-catalyzed directed and undirected, proximal as well as distal pyridine C(sp^2^)–H bond functionalizations in detail under different types of reactions. Further, this review excludes the use of pyridine as a directing group for C–H functionalizations and the C–H functionalization of fused pyridines.

## Review

### C–H Alkylation of pyridine

The C–H bond is the backbone of an organic molecule and the conversion of a C–H bond to a C–X bond (X = carbon or heteroatom) forms the basis in organic synthesis. The functionalization of C–H bonds is challenging due to a large kinetic barrier for C–H bond cleavage and also achieving selectivity is difficult due to its ubiquitous nature [40]. The metal-catalyzed C–H bond functionalization is a good strategy for synthesizing highly functionalized organic frameworks. In this context, the C–H alkylation is one of the most important C–C bond-formation reactions [41–45]. On the other hand, a metal-catalyzed functionalization of arene/heteroarene C–H bonds to the corresponding C–C bonds is an area of great interest and has been well studied [46–47]. Pyridine, being an important heterocyclic scaffold, various studies have been conducted for the C(sp^2^)–H alkylation of the pyridine ring. In this part, we describe pyridine C–H alkylation reactions sub-sectioning based on the position of the alkylation reported.

#### *ortho*-C–H Alkylation

**Scheme 2:**
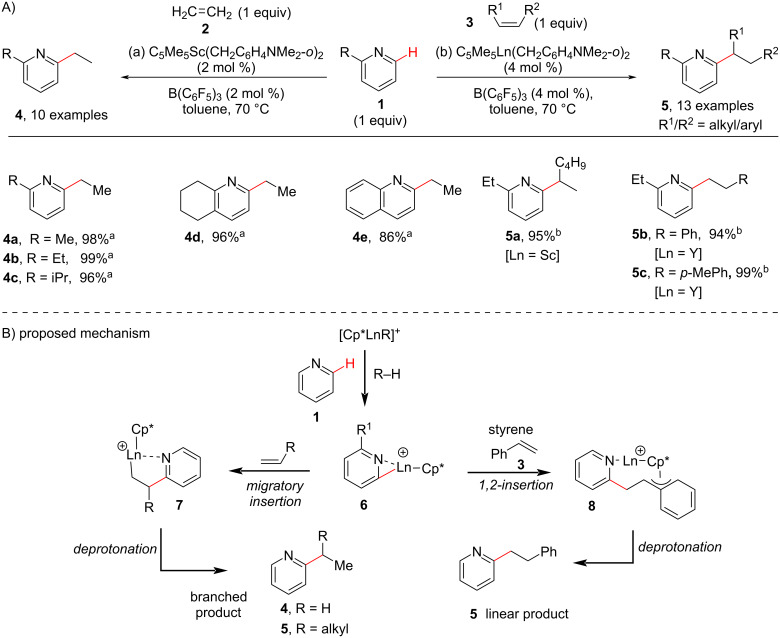
Rare earth metal (Ln)-catalyzed pyridine C–H alkylation.

Inspired by the pioneering work of Jordan and co-workers [48] on the *ortho*-selective C–H alkylation of 2-picoline with propene using a cationic zirconium complex under a H_2_ atmosphere in 1989 and the work done by Bergman and Ellmann [49] in 2010 for the *ortho*-C–H alkylation of pyridines under Rh(I) catalysis at high temperature, in 2011 Hou and Guan reported an atom economical method for the selective *ortho*-alkylation of pyridines by C–H addition to olefins under cationic half-sandwich rare-earth catalysis [50]. They carried out the reaction in the presence of dialkyl complexes of scandium (Sc) or yttrium (Y) such as (C_5_Me_5_)Ln(CH_2_C_6_H_4_NMe_2_-*o*)_2_ (Ln = Sc, Y) in combination with B(C_6_F_5_)_3_ as an activator. The method demonstrated a wide substrate scope of both pyridines and olefins including α-olefins, styrenes, and conjugated dienes. The yttrium complex was found to be superior as compared to the scandium complex for the alkylation reaction of bulkier 2-*tert*-butylpyridine with ethylene. In addition, the yttrium catalyst was also found to have a higher catalytic activity for the *ortho-*alkylation of pyridines with styrenes to give the linear alkylated products (**5b**,**c**, Scheme 2). Further, the authors proposed that the C–H bond activation could be the rate limiting step based on kinetic isotope experiments (KIE). The proposed mechanism involves the coordination of pyridine to the metal center of the cationic catalyst and B(C_6_F_5_)_3_ promotes the *ortho*-C–H activation (deprotonation) of pyridine to afford pyridyl species **6**. Next, the 2,1-migratory insertion of alkene **2** into the metal–pyridyl bond in **6** gives the intermediate **7**, which on subsequent deprotonation gives the branched alkylated product **4**. Whereas, in case of styrene **3** a 1,2-insertion takes place possibly due to the formation of the stable benzallylic species **8**, which on deprotonation gives the linear alkylated product **5**.

**Scheme 3:**
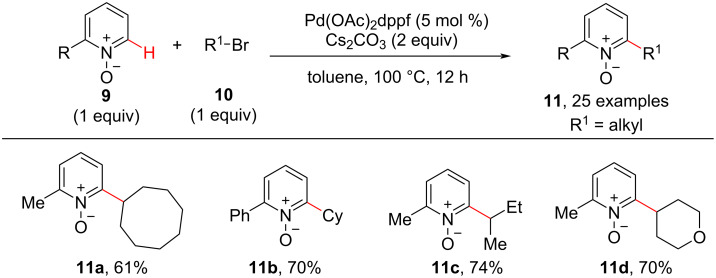
Pd-catalyzed C–H alkylation of pyridine *N*-oxide.

**Scheme 4:**
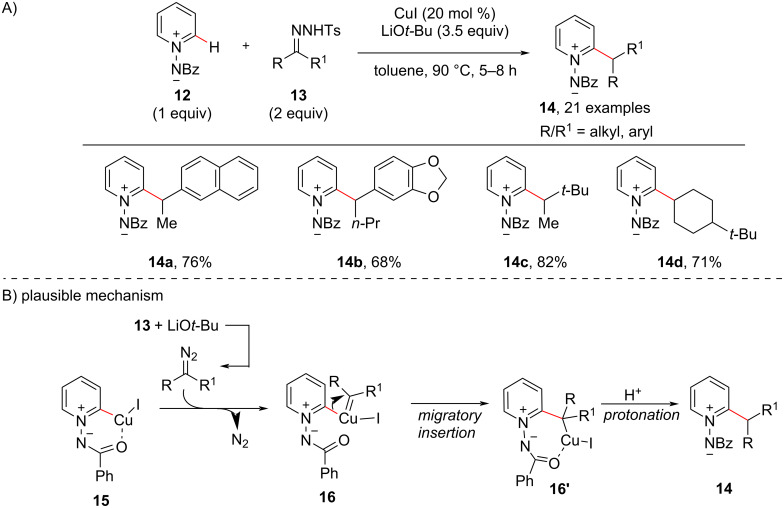
CuI-catalyzed C–H alkylation of *N*-iminopyridinium ylides with tosylhydrazones (A) and a plausible reaction mechanism (B).

The C–H activation/C–C cross-coupling reaction with 1° alkyl electrophiles has been known in the past, however, the C–H alkylation with nonactivated secondary (2°) alkyl electrophiles and tertiary alkyl electrophiles was little known. In this context, in 2013, Fu and co-workers came across an unexpected finding with Pd-catalyzed C–H activation/C–C cross-coupling of pyridine *N*-oxides with nonactivated secondary (2°) alkyl bromides [51]. The cross-coupling is difficult to achieve as the Pd-catalyzed S_N_2 process is sensitive towards the steric bulk of the secondary or tertiary alkyl electrophiles. The optimized conditions for the *ortho*-alkylation of pyridine *N*-oxides **9** with nonactivated secondary (2°) alkyl bromides **10** required 5 mol % of the Pd(OAc)_2_dppf catalyst, Cs_2_CO_3_ (2.0 equiv) as base in toluene at 100 °C as shown in Scheme 3. Under these conditions, the reaction provided diverse 2-alkylpyridine derivatives **11** in moderate to good yields starting from both cyclic and acyclic alkyl bromides. The findings of the reaction’s stereochemistry and observations made during some cyclization or ring-opening reactions indicated that the C–H alkylation may proceed through a radical-type mechanism. Next, in 2013, Wang and co-workers [52] reported a protocol using CuI (10 mol %) as inexpensive catalyst and LiO*t*-Bu (3.5 equiv) as the base for the C–H alkylation of *N*-iminopyridinium ylides **12** with *N*-tosylhydrazones **13** showing good substrate scope for both coupling partners (Scheme 4). A substituent on the aromatic ring of the tosylhydrazones did not significantly affect the C–H alkylation reaction and the reaction also proceeded well with hydrazones **13** obtained from aliphatic aldehydes or ketones. Based on mechanistic experiments and DFT calculations, the reaction presumably proceeds via a Cu–carbene migratory insertion (Scheme 4b). In the presence of CuI and the base the initial direct C–H activation of the ylide **12** gives the copper pyridinium ylide **15**. The latter reacts with the diazo compound formed through reaction of hydrazone **13** with the base to give the copper–carbene species **16**. Then, the intermediate **16** undergoes a Cu–carbene migratory insertion giving intermediate **16’**, which upon protonation gives the desired alkylated product **14**.

**Scheme 5:**
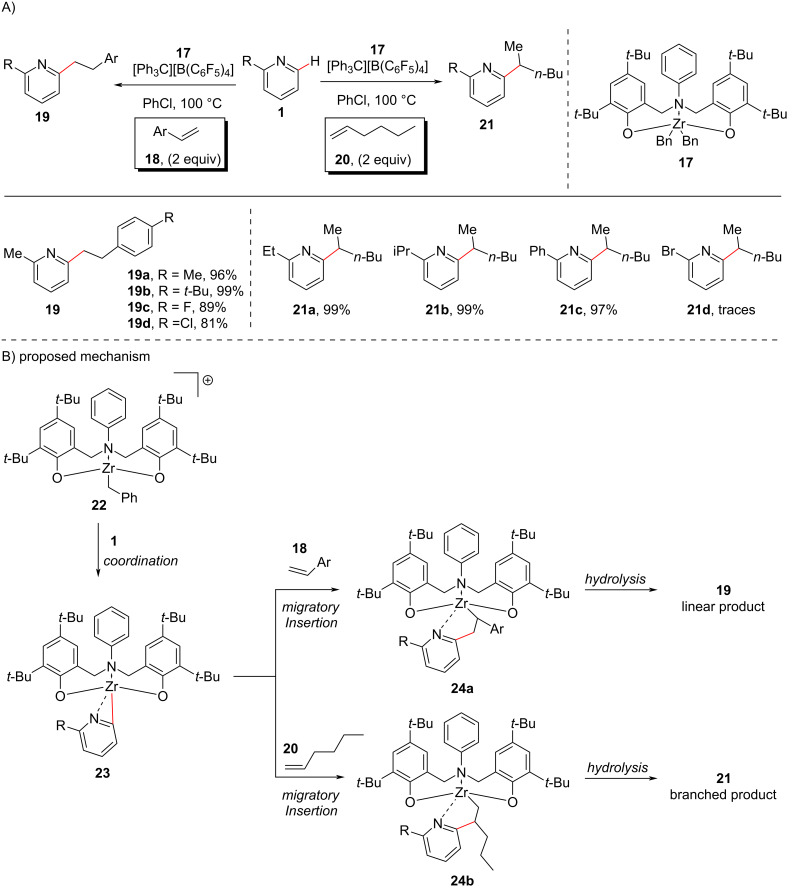
Zirconium complex-catalyzed pyridine C–H alkylation.

Later, in the year 2018, Yao and co-workers [53] developed the first example of a group 4 metal-based catalyst protocol for the C–H alkylation of pyridine **1** with alkenes **18** and **20** as coupling partners. They demonstrated that the reaction in the presence of cationic zirconium complexes derived from zirconium dibenzyl complexes bearing tridentate [ONO]-type amine-bridged bis(phenolato) ligands and [Ph_3_C][B(C_6_F_5_)_4_] (Scheme 5), gave rise to *ortho*-selective C–H alkylated pyridines **19** and **21**. It was observed that the cationic Zr complexes provided good transformations, probably due to good accessibility of the coordination site and an increased Lewis acidity of the metal center. The authors also demonstrated that this catalytic system also catalyzes the alkylation of benzylic C–H bonds (C(sp^3^)–H) of various dialkylpyridines with alkenes. It is to be noted that the ligands’ backbones were found to be crucial for the regioselectivity of the addition to benzylic C(sp^3^)–H bonds, as *N*-arylamine-bridged bis(phenolato) Zr complexes provided branched products whereas *N*-alkylamine-bridged bis(phenolato) Zr complexes provided the linear addition products. The proposed mechanism (Scheme 5b) involves the initial formation of Zr complex **22** through the reaction of neutral Zr complex **17** with [Ph_3_C][B(C_6_F_5_)_4_], which on coordination with the pyridine resulted in the formation of the 3-membered zirconacyclic intermediate **23**. The migratory insertion of the alkene into the metal–C bond of **23** gives the intermediate **24a** on reaction with styrene **18** and intermediate **24b** in the presence of alkene **20**. The intermediates **24a** and **24b** then undergo further hydrolysis to give the desired linear products **19** and branched products **21**, respectively.

**Scheme 6:**
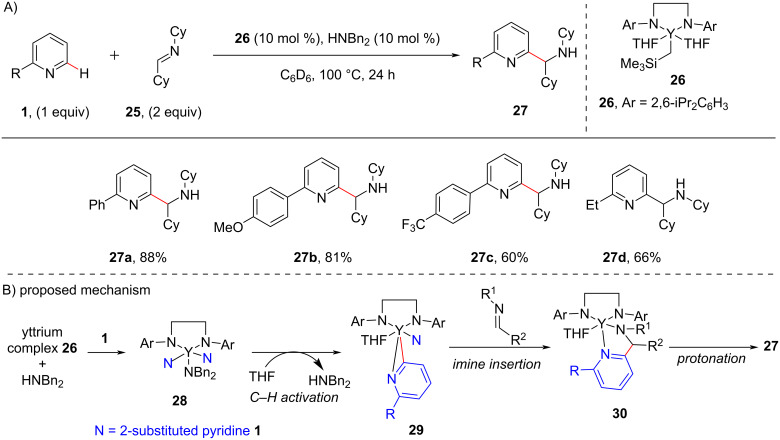
Rare earth metal-catalyzed pyridine C–H alkylation with nonpolar unsaturated substrates.

In the same year, Tsurugi and Mashima reported the use of rare earth metal complexes for the insertion of nonpolar unsaturated substrates (C=N) into the *ortho*-C–H bond of pyridine derivatives [54]. They carried out the C–H aminoalkylation of pyridines **1** using yttrium complex **26** with nonactivated imines **25** (Scheme 6). The authors also demonstrated the enantioselective aminoalkylation, using chiral diamines as ligands. The introduction of chiral diamines in the metal complex produced the aminoalkylated products enantioselectivity with good ratio of enantiomeric excess. The plausible mechanism involves the formation of (dibenzylamido)yttrium complex **28** by the reaction of yttrium complex **26** with HNBn_2_. Then σ-bond metathesis between the Y–N bond of **28** and the *ortho*-C–H bond of pyridine gives η^2^-pyridyl species **29** which on imine insertion produces species **30**. Subsequent protonation then provides the aminoalkylated product **27** (Scheme 6b).

**Scheme 7:**
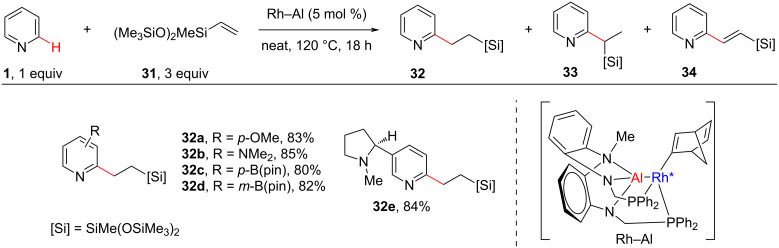
Heterobimetallic Rh–Al complex-catalyzed *ortho-*C–H monoalkylation of pyridines.

The selective C–H monoalkylation of pyridines with alkenes is a challenging task. Most *ortho*-C–H alkylation reactions have been achieved starting from C2-substituted pyridines. There are a few studies reported for the selective C–H monoalkylation of unsubstituted pyridines, which, however, displayed limited substrate scope [55–56]. In this regard, in 2021, Nakao and co-workers [57] reported a selective C2-monoalkylation of 2,6-unsubstituted pyridines with alkenes **31** using a heterobimetallic Rh–Al catalyst. The reaction provided the linear product **32** with aliphatic alkenes **31**, whereas vinylarenes produced the branched product **33** and also alkenylated products **34**. The reaction gave excellent yields of the *ortho*-alkylated products with good functional group tolerance (Scheme 7).

**Scheme 8:**
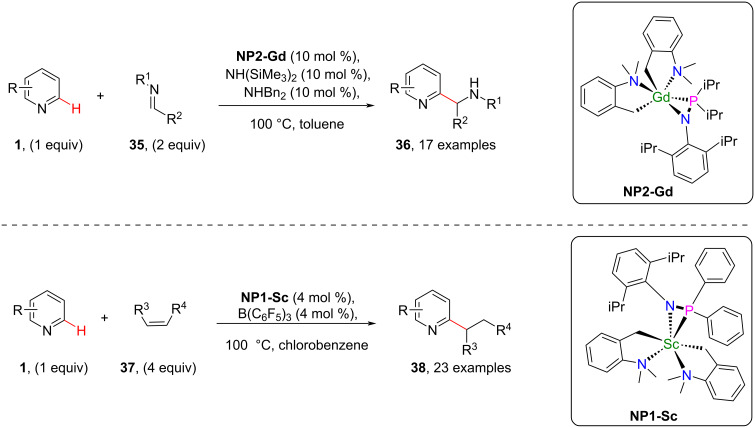
Mono(phosphinoamido)-rare earth complex-catalyzed pyridine C–H alkylation.

The C–H functionalization of pyridines through action of different catalyst systems including transition metals and rare earth metals has been described and some other organometallic systems also were shown to have catalytic reactivity. Adopting this catalytic reactivity of organometallics and also the special bidentate nature of phosphinoamide ligands, in 2021, Chen and group [58] described the catalytic *ortho*-C(sp^2^)–H functionalization of pyridines with polar imines **35** and nonpolar alkenes **37** by using mono(phosphinoamido)-ligated rare earth complexes (**NP2-Gd** and **NP1-Sc**) as shown in Scheme 8. Complex **NP2-Gd** was found to be effective in the functionalization of pyridines with imines providing various *ortho*-aminoalkylated products **36** whereas *ortho*-alkylated pyridine derivatives **38** were obtained when using **NP1-Sc** as the catalyst (Scheme 8).

**Scheme 9:**
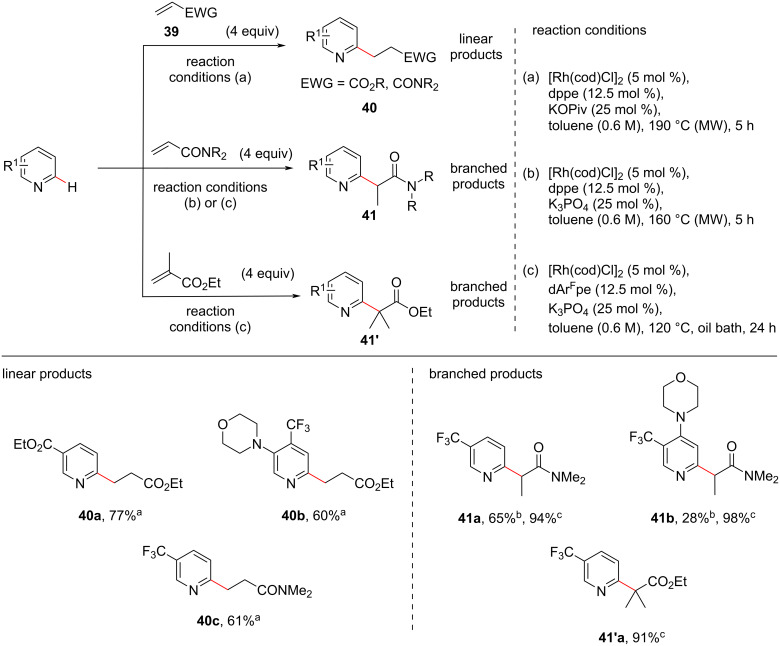
Rhodium-catalyzed pyridine C–H alkylation with acrylates and acrylamides.

Attributing to the strong coordination of unsubstituted pyridine with Rh(I) catalysts, C–H alkylations of pyridine lacking *ortho*-blocking groups is a challenge. In this context, a regioselective alkylation of *ortho*-unsubstituted or substituted unactivated pyridines with acrylates and acrylamides under Rh(I) catalysis has been demonstrated by Ellman and co-workers [59]. The authors observed that in the presence of [Rh(cod)Cl]_2_ as catalyst, dppe as ligand, and potassium pivalate (KOPiv) as base, linear C–H-alkylated products **40** were obtained from both acrylates and acrylamides in moderate to high yields (Scheme 9, reaction conditions a). However, when K_3_PO_4_ was employed as the base under otherwise identical conditions, the authors observed a switch in regioselectivity and branched products **41** were obtained with acrylamides as coupling partners (Scheme 9, reaction conditions b). Thus, the authors demonstrated a switch in regioselectivity (linear/branched) which was controlled exclusively by the base used. During further investigations the authors found that the use of ligand dArFpe at reduced reaction temperature resulted in a significant increase in the yield of the branched alkylated product **41** (Scheme 9, reaction conditions c) compared to using the ligand dppe (Scheme 9, reaction conditions b). Moreover, when ethyl methacrylate was used as the coupling partner under the reaction conditions c, branched alkylated products **41’** were obtained selectively in high yields (Scheme 9). A high functional group tolerance was observed in both linear and branched alkylated products.

**Scheme 10:**
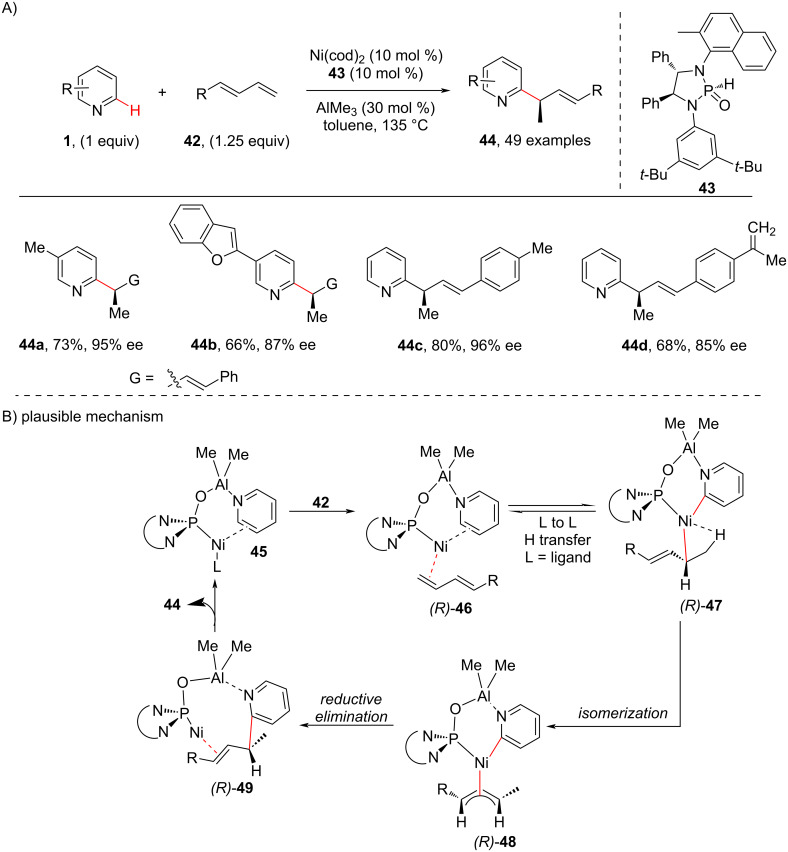
Ni–Al bimetallic system-catalyzed pyridine C–H alkylation.

It is known that the strong coordination of the nitrogen atom in pyridine rings with metals inhibits the metal–chiral ligand coordination, thus making the C–H alkylation of pyridine substrates challenging. In addition, transition-metal-catalyzed enantioselective C–H alkylation reactions of pyridine still remain a great challenge. In this regard, in 2022, Ye and co-workers [60] reported for the first time an enantioselective C-2 alkylation of pyridine using a chiral phosphine oxide-ligated Ni–Al bimetallic catalyst system and the protocol was found effective for a wide range of pyridines including unsubstituted pyridines, C2, C3 and C4-substituted pyridines and complex pyridines containing bioactive molecules (Scheme 10). To attain enantioselectivity a chiral phosphine oxide (**43**)-ligated Ni–Al bimetallic catalyst was used that was critical in improving the reactivity and controlling the selectivity of the reaction. Further, based on deuterium labelling experiments, KIE studies, and DFT calculation, a plausible mechanism (Scheme 10b) has been proposed. Initially, a reversible ligand-to-ligand H-transfer process occurs for C–H activation between the intermediates **46** and **47**. Next, isomerization of the η^1^-allyl complex **47** forms the η^3^-allylic nickel complex **48**, which on reductive elimination delivers the desired product **44** via the intermediate **49** (Scheme 10b). It was proposed that the enantioselectivity was mainly due to the C–C reductive elimination of the *R*-pathway, which is lower in energy than the *S*-pathway.

#### Remote C–H alkylation

**Scheme 11:**
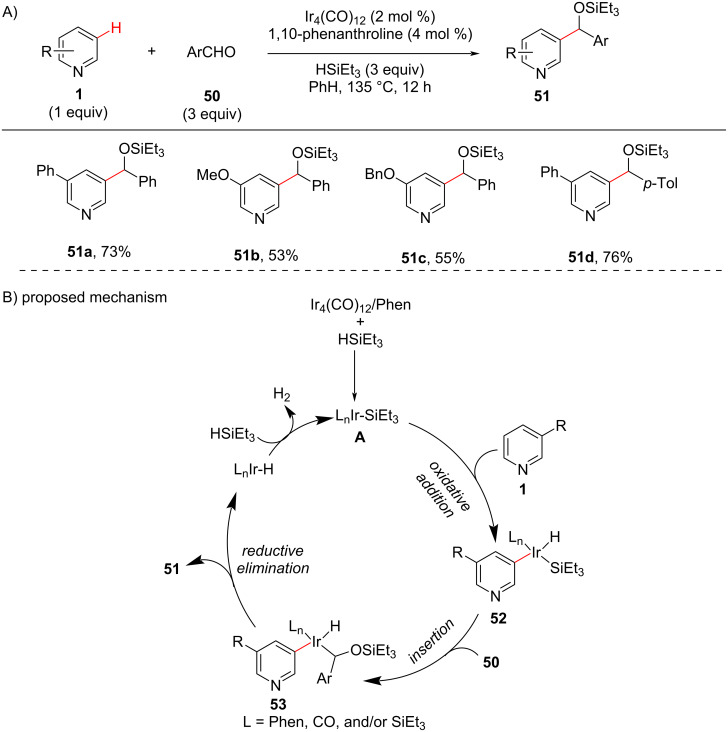
Iridium-catalyzed pyridine C–H alkylation.

Several remarkable studies have been reported for proximal C–H functionalizations in pyridine substrates under different catalytic systems. However, the intermolecular undirected distal C–H functionalization in pyridine remained unstudied. In these circumstances, the distal C–H alkylation by addition of the pyridine C–H bond to an aldehyde **50** under iridium catalysis was achieved by Shi [61] in 2010 through an unusual *meta*-selectivity for the first time (Scheme 11a). To achieve *meta*-selectivity, the group has screened various transition metals and revealed that a silyl-iridium complex promoted the addition of *meta*-pyridyl C–H bonds to aldehydes **50** which resulted in C3-alkylated pyridines **51**. Based on the reactions performed for the catalytic activity of the silyl-iridium complex, the authors proposed a catalytic mechanism (Scheme 11b). The mechanism involves the initial formation of the active silyl-iridium catalyst **A** which through oxidative addition of **1** gives the silyl-iridium complex **52**. The insertion of aldehyde **50** into the Ir–Si bond of **52** provides the pyridyl alkyl iridium species **53** that finally by C–C formation via reductive elimination furnishes the desired products **51** along with the formation of an iridium hydride species (Scheme 11b).

**Scheme 12:**
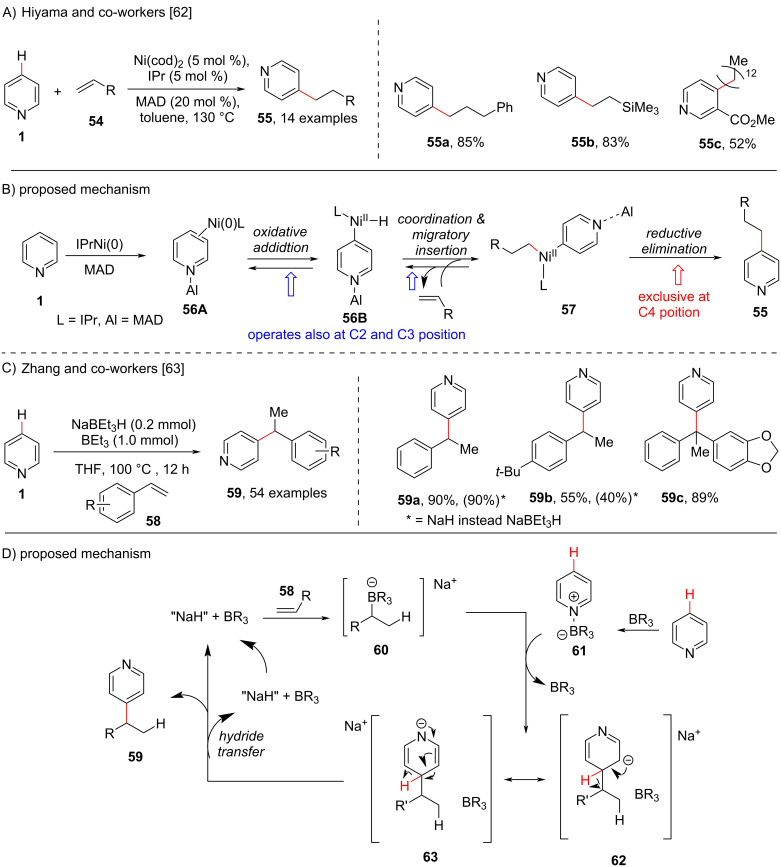
*para*-C(sp^2^)–H Alkylation of pyridines with alkenes.

A direct selective C4-alkylation of pyridine has been reported by the groups of Hiyama [62] (Scheme 12a) and Zhang [63] (Scheme 12c) in 2010 and 2020, respectively. The Hiyama group developed a C-4-selective alkylation of pyridines using a Ni/Lewis acid cooperative catalytic system in combination with a bulky N-heterocyclic carbene ligand and (2,6-*t*-Bu_2_-4-Me-C_6_H_2_O)_2_AlMe (MAD) as the Lewis acid which allowed the direct C-4 alkylation of pyridines **1** (Scheme 12a). With the optimized reaction conditions in hand the group also screened the alkene and pyridine substrate scope which resulted C4-alkylated products **55** in moderate to high yields. A possible mechanistic cycle (Scheme 12b) was also proposed, comprising an initial formation of η^2^-arenenickel species **56A**, which undergoes oxidative addition to the C(4)–H bond of pyridine to form intermediate **56B**. Next, coordination and migratory insertion of the alkene provides the intermediate **57** which on subsequent reductive elimination furnishes the C4-alkylated products **55**. Based on the deuterium exchange experiment, the author suggested that the steps involved in the catalytic cycle from **56A** to **57** are reversible in nature, which may activate the C2 or C3 position as well. However, the reductive elimination at the C4-position was suggested to be irreversible in nature and does not takes place at the C2 and C3 position. On the other hand, the Zhang group reported the C4 alkylation of pyridines using alkenes **58** catalyzed by an organoborohydride (NaBEt_3_H) and aided by organoboranes (Scheme 12c). The proposed mechanism (Scheme 12d) involves the formation of the organoborate intermediate **60** from alkene **58** in the presence of the NaBEt_3_H catalyst and the organoborane. Next, the organoborane-activated pyridine species **61** undergoes an addition reaction regioselectively at the C4 position of the organoborate intermediate **60** delivering the σ^H^-adduct intermediates **62** and **63**. Subsequently, hydride elimination with the help of the organoborane gave the desired alkylated product **59** and regenerates the hydride catalyst.

**Scheme 13:**
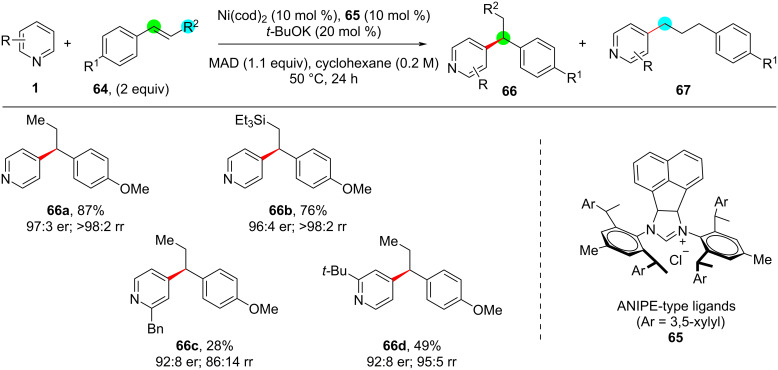
Enantioselective pyridine C–H alkylation.

Further enantioselective pyridine C–H alkylation reactions are very scarcely reported which specifically include the intramolecular C–H alkylation of pyridine with alkenes at the C3 or C4 positions. Hence, very recently in 2022, Shi and co-workers [64] adopted an intermolecular process and reported the enantioselective *para*-alkylation of pyridines with styrenes **64** using a Ni–Al bimetallic system and NHC ligand **65** through intermolecular hydroarylation with high levels of enantio- and regioselectivity in the alkylated products **66** (Scheme 13). Also, the authors performed DFT studies revealing the reaction mechanism and supported that the interaction of the NHC aryl part with *trans*-styrene was highly important for the reaction to proceed and for the enantiocontrolled formation of the products.

### Alkenylation

The C–H alkenylation is another important C–C bond-forming reaction. Olefinated organic molecules like vinylarenes play a significant role as key intermediates in organic synthesis and are also present in various natural products as well as drug molecules [65–68]. Though there are traditional methods available for C–H olefinations they suffer from some disadvantages such as for example requiring prefunctionalized substrates as in case of the Heck cross-coupling [69–70]. However, researchers have developed various methods for the transition-metal-catalyzed C(sp^2^)–H olefination using various types of alkenes as coupling partners [71–73]. This part of the review covers reports for the alkenylation of pyridine with terminal alkynes, acrylates, allenes, and alkynes as coupling partners achieving the functionalized C(sp^2^)–H-olefinated pyridine frameworks via metal catalysis.

#### *ortho*-C–H Alkenylation

**Scheme 14:**
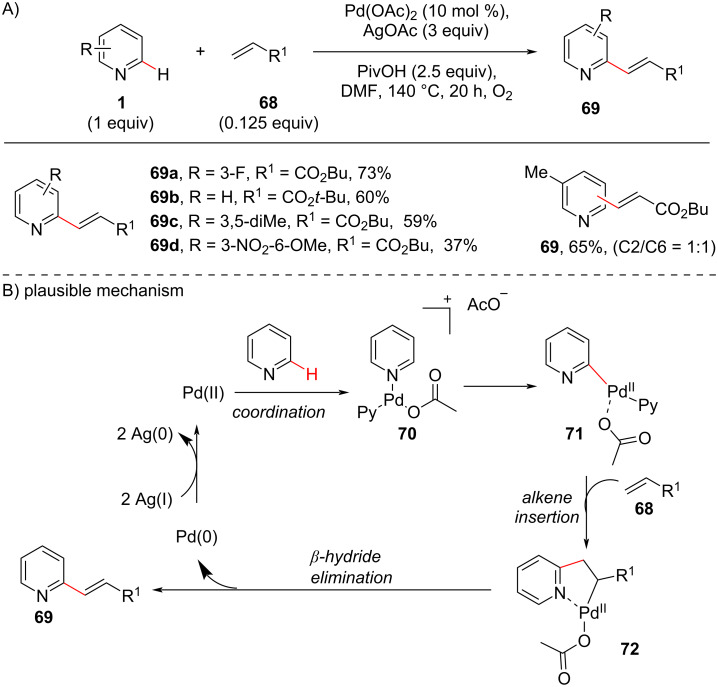
Pd-catalyzed C2-olefination of pyridines.

In 2012, Huang and co-workers [74] disclosed a ligand-free oxidative cross-coupling reaction of pyridine with acrylates, acrylamides, and styrenes (Scheme 14). Their preliminary investigation provided both C2 and C3-olefinated products, with the C2-selective product **69** as the major product (Scheme 14a). With the optimized conditions of Pd(OAc)_2_ (10 mol %), AgOAc (3 equiv), PivOH (2.5 equiv) in DMF, the method showed wide substrate scope and good yields. Based on the experimental findings the authors proposed a catalytic cycle (Scheme 14b) which commences with the coordination of Pd(II) with the pyridine nitrogen to provide intermediate **70**. A strong *trans*-effect results in the C–H cleavage for the formation of Pd(II) species **71**. Subsequently, insertion of alkene **68** provides the cyclic Pd(II) intermediate **72** which undergoes β-hydride elimination to produce the desired product **69**.

**Scheme 15:**
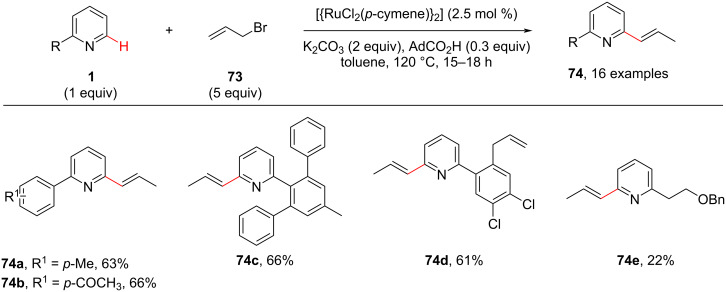
Ru-catalyzed C-6 (C-2)-propenylation of 2-arylated pyridines.

In the same year, Ramana and Goriya [75] proposed an unexpected C-6 (C-2)-propenylation reaction of pyridine in the presence of allyl bromide (**73**) and a Ru catalyst using 2-arylpyridines (Scheme 15). Earlier reports described the propenylation took place on the *ortho*-position of the phenyl ring [76–77], whereas this group achieved the propenylation of the pyridine moiety. The authors screened different allyl halides and Ru complexes as catalysts. With the optimized conditions in hand, diverse 2-arylated pyridines were screened resulting in the corresponding products **74** in good yields.

**Scheme 16:**
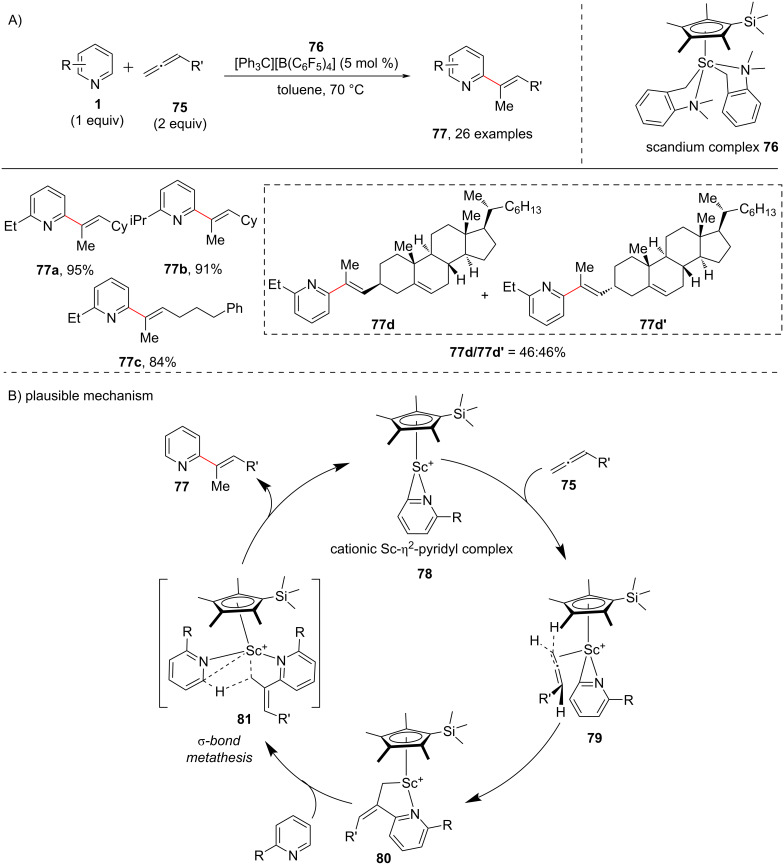
C–H addition of allenes to pyridines catalyzed by half-sandwich Sc metal complex.

Allene, a cumulated diene and an important building block in organic synthesis has versatile biological properties and is also an important subunit in various natural products and pharmaceutical compounds [78]. Allenes have been applied as useful substrates for the alkenylation of organic molecules [79]. There are various reports for the C–H alkenylation of aromatic C–H bonds using allenes [80]. To this end, Hou and group in 2015 [81] demonstrated the C–H allenylation of pyridines with excellent substrate scope using a scandium catalyst (Scheme 16). A vast number of pyridines and allenes were studied as substrates to provide the C2-alkenylated pyridines in good to high yields. Based on the mechanistic experiments a possible catalytic cycle has been proposed (Scheme 16b). The half-sandwich scandium complex **76** along with the tetrakis(pentafluorophenyl)borate and pyridine forms a cationic Sc-pyridyl complex **78**, which after addition of allene **75**, forms a transient pentacyclic intermediate **80** via intermediate **79**. Next, another molecule of pyridine adds to intermediate **80** to furnish the transient complex **81** which undergoes σ-bond metathesis to give the product **77** and regenerating **78** (Scheme 16b).

**Scheme 17:**
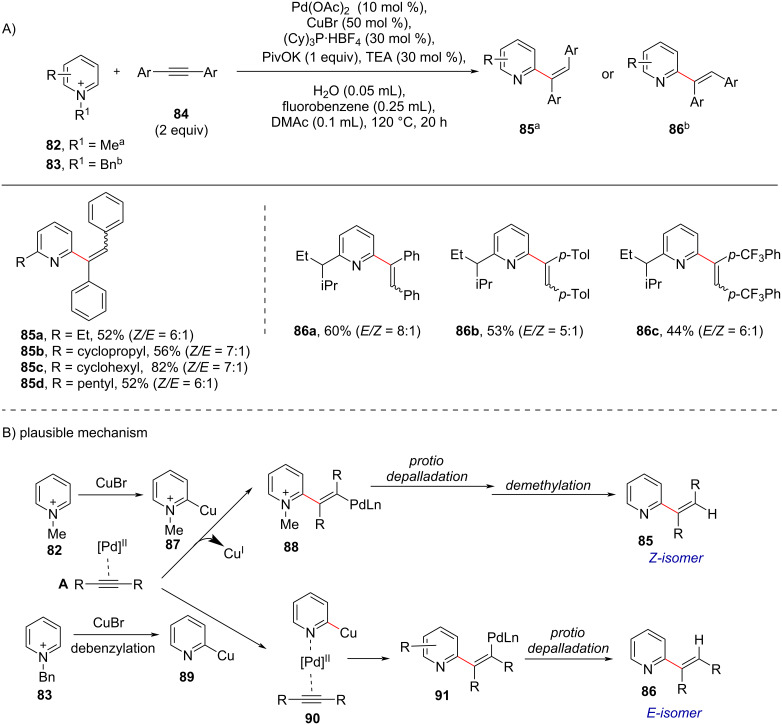
Pd-catalyzed stereodivergent synthesis of alkenylated pyridines.

While speaking regarding the alkenylation, the geometrical isomerism, i.e., the stereoselectivity between the *cis*- and *trans*-alkenylation, has not been considered so far. Except lately, in 2020, Chen and group [82] reported a Pd/Cu-catalyzed regio- and stereoselective synthesis of C2-alkenylated pyridines starting from internal alkynes **84** and pyridinium salts in a stereodivergent manner (Scheme 17a). The interesting part of this work was the switching of the alkene configuration of the products by modifying the substituents on the nitrogen of the pyridinium salts. Further, the method showed a wide substrate scope for both the *Z*- and *E*-alkenylated products in which *Z*-selectivity was achieved when *N*-methylpyridinium salts were used and *E*-selectivity when *N*-benzylpyridinium salts were used.

In the proposed mechanism (Scheme 17b) the *E-* and *Z*-isomers can be assessed through point at which dealkylation occurs, i.e., if it occurs as last step the *Z*-isomer **85** is obtained and if it takes place at an early stage, *E*-isomer **86** predominates (Scheme 17b). The proposed mechanism involves the initial formation of π-complex **A** via activation of the alkyne by Pd. Then, in case of *N*-methylpyridinium salt **82**, in presence of CuBr the pyridine–Cu(I) complex **87** is formed through C–H activation that further undergoes nucleophilic attack to the coordinated alkyne in a *trans*-manner to give Pd(II)–alkenyl intermediate **88**. Then, the intermediate **88** undergoes protio-depalladation and demethylation to yield the *Z*-isomer **85** (Scheme 17b). In case of *N*-benzylpyridinium salts **83**, first debenzylation occurs to form 2-pyridyl–Cu(I) species **89** in the presence of CuBr which then coordinates to the Pd center of π-complex **A** via the lone electron pair of the pyridine nitrogen to give **90** which further attacks the π-bond in a *cis*-manner to give intermediate **91**. After protio-depalladation the *E*-isomer **86** is obtained as major product (Scheme 17b).

#### Remote alkenylation

**Scheme 18:**
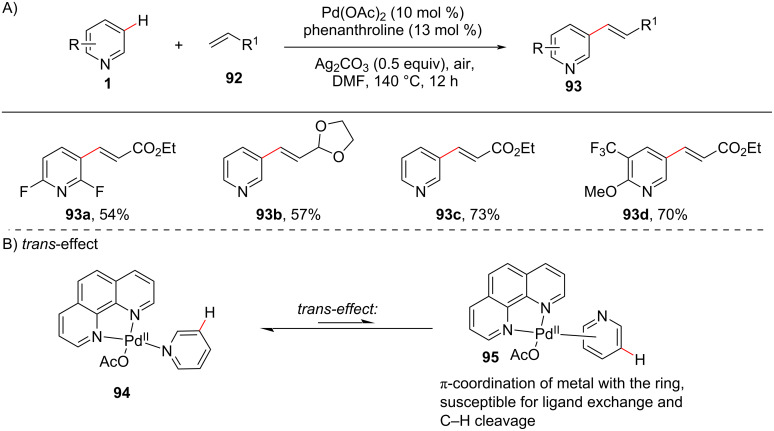
Pd-catalyzed ligand-promoted selective C3-olefination of pyridines.

In 2011, a study for weakening the strong coordination of the pyridyl *N*-atom with Pd in the presence of a bidentate ligand was reported by Yu and co-workers [83]. They showcased the C3-selective olefination of pyridines using 1,10-phenanthroline, a bis-dentate ligand that weakens the coordination of the Pd catalyst with the pyridyl *N*-atom through the *trans*-effect (Scheme 18). The *trans*-effect is the switching of the metal coordination between the π-ring system and the hetero-(nitrogen) atom of pyridine [84–85]. In comparison to coordination with nitrogen, which is strong in nature, the coordination with the ring is weaker and cleavable. The usage of a bidentate ligand will enhance the *trans*-effect and shift the coordination towards the ring (Scheme 18b).

**Scheme 19:**
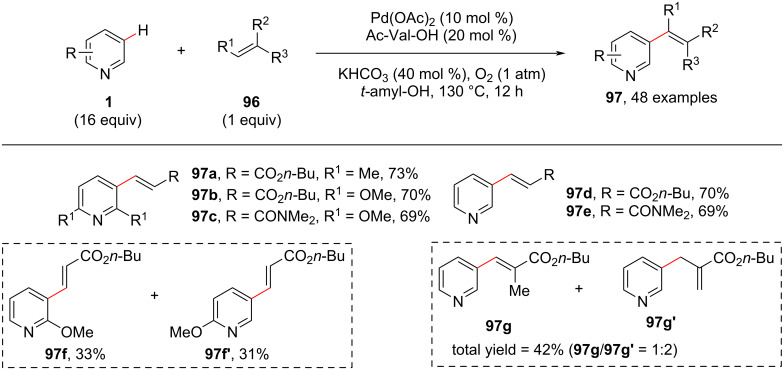
Mono-*N*-protected amino acids in Pd-catalyzed C3-alkenylation of pyridines.

There are numerous studies reported for directing group or chelate-assisted metal-catalyzed C–H functionalization reactions. However, non-chelate-assisted or undirected C–H functionalizations under ligand-controlled conditions are underexplored. Hence, differentiating from this co-coordinative model, in 2013, Zeng and co-workers [86] reported the MPAA (mono-*N*-protected amino acids) ligand-promoted non-chelate-assisted C–H activation via Pd-catalyzed dehydrogenative Heck reactions on pyridines with simple alkenes **96**, leading to the C3-alkenylated products **97** (Scheme 19). The reaction was based on the previous reports of using of the MPAA ligands in the Pd-catalyzed oxidative cross-coupling reactions discovered by Yu et al. [87]. When 2-methoxypyridine was screened, the reaction resulted in a mixture of C3- and C5-selective C–H-functionalized products **97f** and **97f'** in a regioisomeric ratio of nearly 1:1. Further, during the substrate scope study, when 1,1′-disubstituted butyl methacrylate was used as coupling partner a mixture of **97g** and the isomeric product **97g'** was observed in 42% yield.

**Scheme 20:**
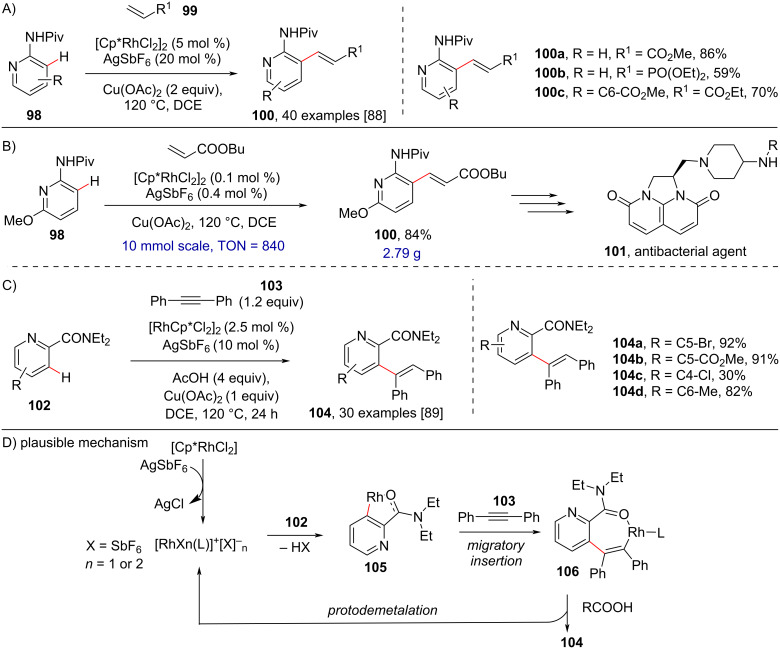
Amide-directed and rhodium-catalyzed C3-alkenylation of pyridines.

Further, Shi and co-workers reported the rhodium-catalyzed directed C–H olefination of pyridines using different directing groups in 2013 [88] (Scheme 20a) and 2014 [89] (Scheme 20c), respectively. In the former study, under optimized conditions of [RhCp*Cl_2_]_2_ (5 mol %), AgSbF_6_ (20 mol %) in DCE at 120 °C, Cu(OAc)_2_ was found crucial for the transformation in comparison to other additives and showed good substrate scope while unactivated alkenes like styrene resulted in no reaction. Also, the authors successfully applied the developed protocol to a multigram-scale synthesis of compound **101**, a tricyclic imidazonaphthyridinone derivative having antibacterial properties, with low catalyst loading (0.1 mol %) (Scheme 20b). Later, in 2014, the same authors, using an amide as directing group (DG), developed a protocol for the regioselective C3-alkenylation of pyridines through *syn*-addition of alkynes, displaying broad substrate scope and high yields (Scheme 20c). Based on literature reports and experimental studies, a possible mechanism (Scheme 20d) was proposed in which coordination of the DG **102** to the rhodium cationic species followed by *ortho*-metalation and migratory insertion of **103** into the Rh–C bond of **105** provides a seven-membered rhodacyclic intermediate **106**. The protonation at the Rh–C bond of intermediate **106** in the presence of RCOOH furnishes hydroarylation product **104**.

**Scheme 21:**
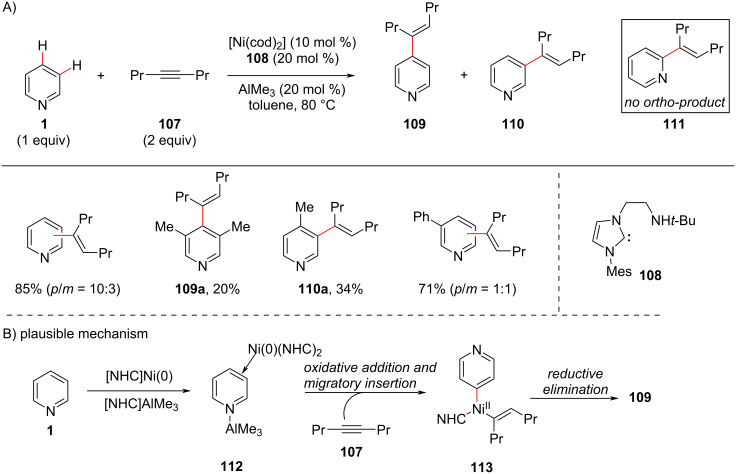
Bimetallic Ni–Al-catalyzed *para*-selective alkenylation of pyridine.

Nitrogen heterocyclic carbenes (NHCs) are of central importance in organometallic chemistry and in organic synthesis. Also, metal–NHC complexes have wide application in catalysis and various organic transformations and a range of metal–NHCs served as catalysts. In 2010, using NHC ligands, Yap and co-workers [90] developed a method for the direct *para* and *meta*-C–H alkenylation of pyridines with 4-octyne (**107**) using a nickel Lewis acid catalyst with amino pendant linked NHC complex (Scheme 21). In addition, the authors were able to isolate the bimetallic intermediate structure η^2^,η^1^-pyridine–Ni(0)–Al(III) complex **112**, as a support for their mechanism for the *para*-C–H functionalization. They further investigated the scope and limitations of the dual catalyst Ni–AlMe_3_ and also the sensitivity of the reaction towards the steric environment on the pyridine ring. The complex **112** undergoes oxidative addition followed by an alkyne insertion reaction to give intermediate **113**, which after reductive elimination provides the alkenylated product **109** (Scheme 21b).

### Arylation

#### C-2 Arylation

**Scheme 22:**
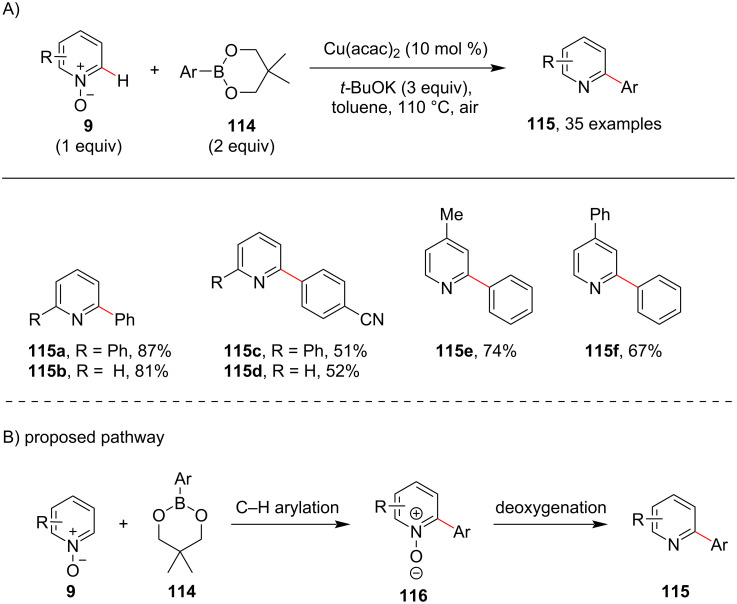
Arylboronic ester-assisted pyridine direct C–H arylation.

Owing to the remarkable role of aromatic C–H arylation reactions in organic synthesis abundant methods have been reported for aromatic C–H arylations using different arylating coupling partners, such as for instance, aryl halides. In 2014, using organoboron coupling partners, Wu and co-workers [91] reported a protocol for the Cu-catalyzed C–H arylation of pyridine *N*-oxides **9** with arylboronic esters **114** and prepared C2-arylated pyridines **115** in moderate to good yields (Scheme 22). By using an inexpensive Cu catalyst, the method allows for the simple and practical synthesis of 2-arylpyridines. The reaction starts with the formation of arylated pyridine *N*-oxide **116** by reaction of pyridine *N*-oxide **9** with the arylboronic ester **114** in the presence of Cu catalyst and base which is followed by deoxygenation to furnish the desired product **115** (Scheme 22b).

**Scheme 23:**
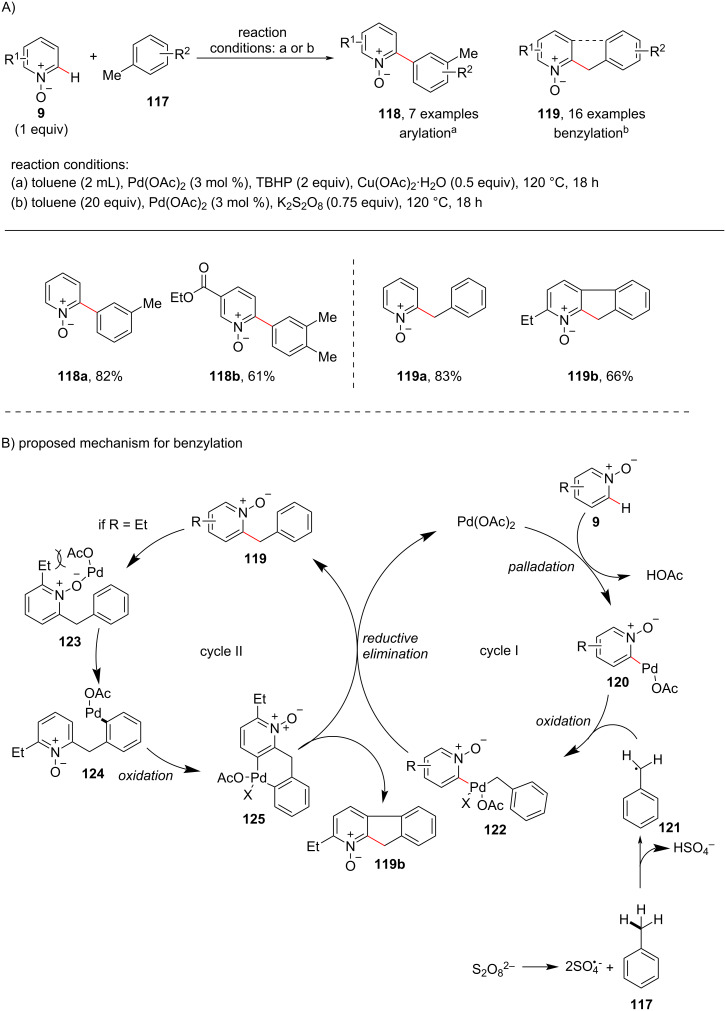
Pd-catalyzed C–H arylation/benzylation with toluene.

In 2015, a palladium-catalyzed cross dehydrogenative coupling of pyridine *N*-oxides with toluene for the regioselective arylation and benzylation of pyridine *N*-oxide was reported by Khan and co-workers [92] (Scheme 23). The authors have shown toluene **117** when used as benzyl and aryl source remained intact under the reaction conditions without any further oxidation. Different oxidants resulted in different products such as the monoarylated product **118** formed in the presence of TBHP as oxidant and the benzylated product **119** was obtained when potassium persulfate was used. Interestingly, aza-fluorene *N*-oxide **119b** was formed during benzylation of 2-ethylpyridine *N*-oxide. A possible mechanism has also been reported (Scheme 23b). Electrophilic palladation at the C2-position of pyridine *N*-oxide **9** provides intermediate **120**. The radical intermediate **121** is generated in situ by H-atom abstraction from toluene **117** by sulfate radical anion. Coordination of intermediate **120** and **121** leads to complex **122** which undergoes reductive elimination to provide product **119**. 2-Ethyl-substituted pyridine *N*-oxides may undergo a dual C–H activation due to the buttressing effect of the ethyl group to produce azafluorene *N*-oxide **119b**.

**Scheme 24:**
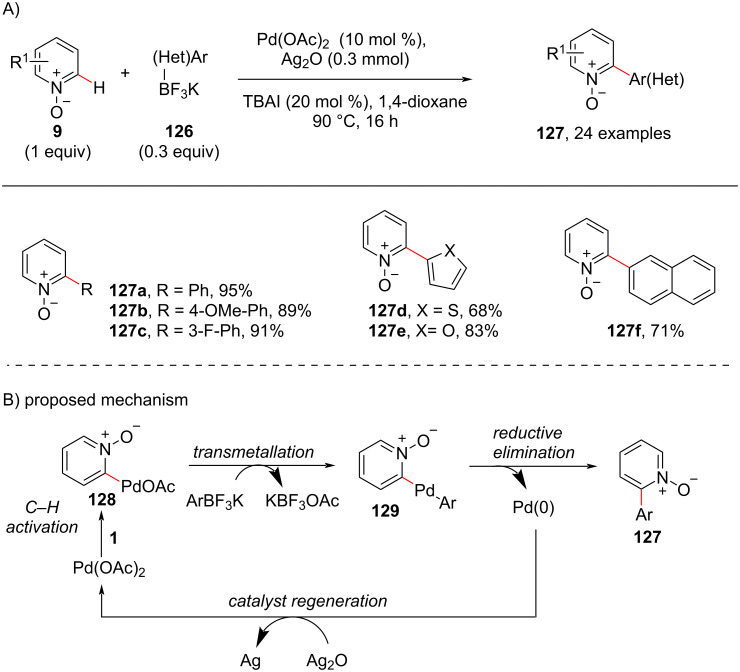
Pd-catalyzed pyridine C–H arylation with potassium aryl- and heteroaryltrifluoroborates.

In 2016, Wei and co-workers [93] reported the arylation of pyridine *N*-oxides **9** employing potassium (hetero)aryltrifluoroborates **126** as coupling partner using palladium acetate and TBAI (Scheme 24). Electron-withdrawing and donating groups on the pyridine *N*-oxide **9** resulted in the corresponding C2-arylated products **127** in good to excellent yields with high site selectivity. A catalytic mechanism was proposed in which the electrophilic C–H palladation of pyridine *N*-oxide **9** occurs preferentially at the C-2 position leading to heterocoupling intermediate **128**. Subsequent transmetalation provides the arylpalladium intermediate **129** which after reductive elimination furnishes the desired product **127**.

**Scheme 25:**
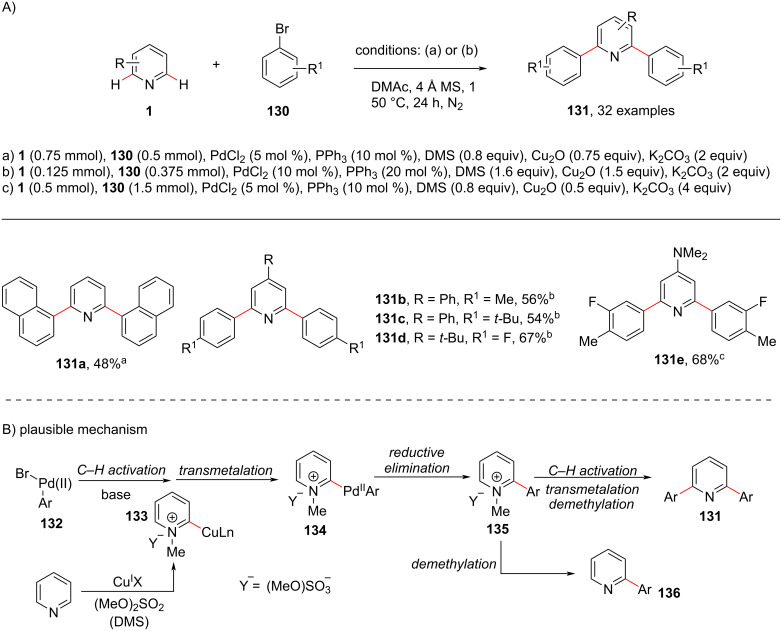
Transient activator strategy in pyridine C–H biarylation.

In 2017, Chen and group [94] developed a protocol for the C2,C6-arylation of pyridine under Pd catalysis (Scheme 25). In their study, *N*-alkylpyridinium salts were used as a directing group, facilitating the C–H arylation of pyridine. Dimethyl sulfate was used as a good *N*-methylating agent, which acts as transient activator. The group performed HRMS and KIE studies and proposed a catalytic cycle (Scheme 25b). The oxidative addition of ArBr **130** to the in situ-formed Pd(0) species gives species **132** followed by transmetalation with CuI pyridyl species **133** generated from the reaction of Cu_2_O with the methylated pyridine to afford intermediate **134** that on reductive elimination results in salt **135**. Subsequent demethylation of **135** gives monoarylated product **136** or the intermediate **135** reenters the catalytic cycle to produce the diarylated *N*-methylpyridinium species, which again undergoes demethylation to produce product **131**.

#### C-3 Arylation

**Scheme 26:**
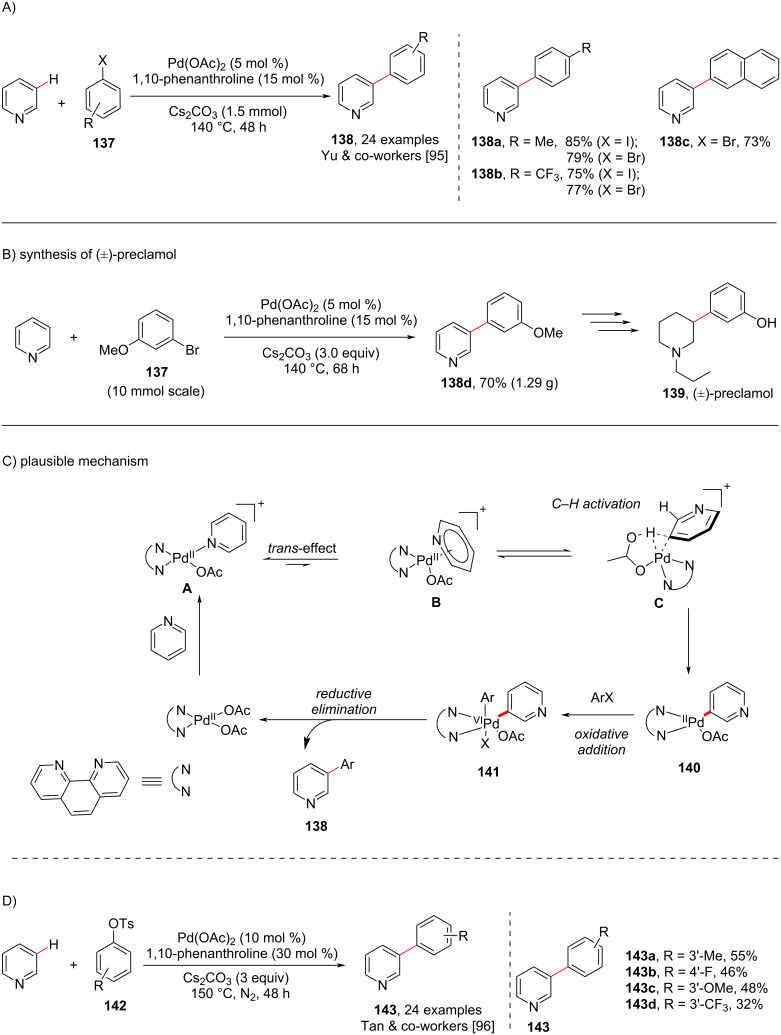
Ligand-promoted C3-arylation of pyridine.

In 2011 and 2013, the groups Yu [95] and Tan [96], reported a ligand-assisted distal arylation selectively taking place at the *meta*-position in pyridine. Both groups used Pd(OAc)_2_ as catalyst with 1,10-phenanthroline as ligand. The group of Yu used aryl halides **137** as coupling partner, whereas the group of Tan utilized aryl tosylates **142** as coupling partner (Scheme 26). The Yu group also applied the developed protocol for the synthesis of the drug molecule preclamol (**139**, Scheme 26b). The presumed catalytic cycle (Scheme 26c) involved the coordination of Pd(II) to the pyridine nitrogen to give *N*-bound pyridine substrate **A** followed by the formation of Pd(II) intermediate (**B**) involving the π-system of pyridine, which initiates the activation of the C(3)–H of pyridine to form aryl–Pd(II) species **140** via intermediate **C**. Subsequently, oxidative addition takes place in the presence of the aryl halide to give the Pd(IV) complex **141** followed by reductive elimination furnishing 3-arylpyridines **138**.

**Scheme 27:**
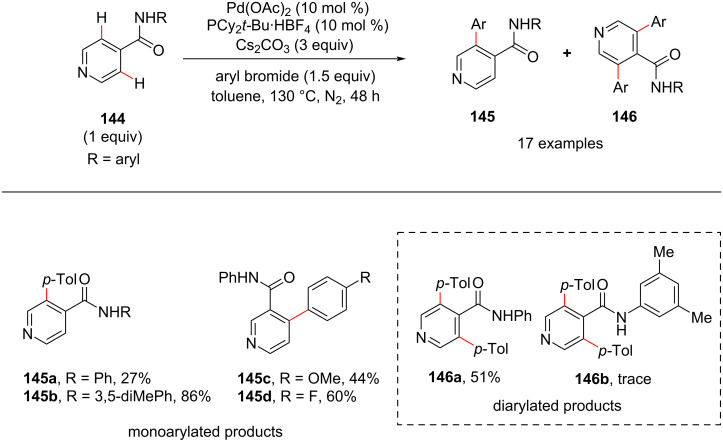
Pd-catalyzed arylation of nicotinic and isonicotinic acids.

Almost at the same time, Yu and co-workers reported the selective Pd(0)/PR_3_-catalyzed C3 or C4-arylation of nicotinic and isonicotinic acids using amide as a directing group (Scheme 27) [97]. This method provides a way for arylated nicotinic acid derivatives which serve as building blocks for biologically important molecules. This was the first report for a directing group-assisted C3/C4-arylation of pyridines. The authors screened various *N*-arylamide directing groups **144** out of which *N*-phenylamide was found to be the better directing group. Then, the authors screened various nicotinic and isonicotinic acids which afforded the desired products **145** and **146** in good yields generating a library of isonicotinic and nicotinic acid derivatives.

**Scheme 28:**
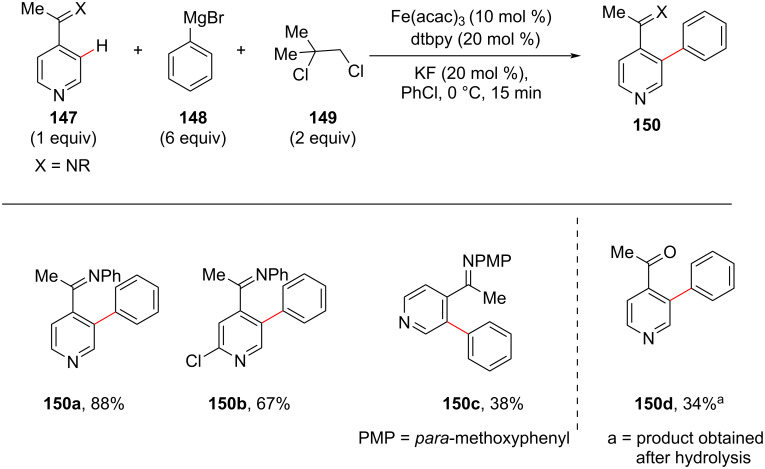
Iron-catalyzed and imine-directed C–H arylation of pyridines.

Another inexpensive and non-toxic iron-catalyzed C–H arylation of pyridines has been reported by DeBeof and co-workers [98]. Using the imine in **147** as directing group, afforded the arylated pyridine products **150** in good to high yields (Scheme 28). In this reaction, Grignard reagent **148** was used as arylation source in excess amount as the reagent underwent homocoupling leading to the formation of biaryl systems under the reaction conditions. 1,2-Dichloro-2-methylpropane (**149**) was found to be an effective oxidant under the reaction conditions. Also, the additive KF was employed in order to minimize the oxidative iron-catalyzed homocoupling of **148**. An imine directing group at the *para*-position in pyridine **147** lead to activated *ortho*-position products **150** within 15 minutes. The imine group of the products can further be hydrolyzed to get the corresponding ketones.

**Scheme 29:**
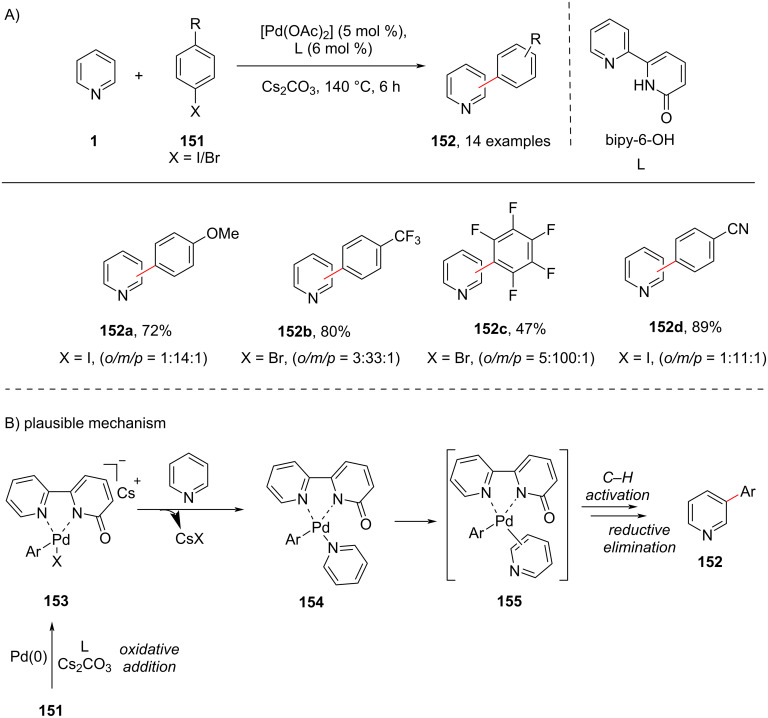
Pd–(bipy-6-OH) cooperative system-mediated direct pyridine C3-arylation.

In 2018, Albéniz and group [99] reported the direct C3-arylation of pyridines with the help of bipy-6-OH as coordinating ligand under palladium catalysis (Scheme 29). In most of the cases the arylated pyridines **152** were obtained as mixtures of *ortho-*/*meta-*/*para*-substitution, however, the authors found that the yield of the *meta* (C-3)-arylated pyridines were drastically higher, thereby showcasing the regioselectivity of the reaction. The chelating anionic ligand acted as base in the catalytic cycle, allowing for the oxidative addition of the arene to the Pd complex. The proposed mechanism (Scheme 29b) involves the oxidative addition of the aryl halide to the Pd(0) complex in the presence of base ligand to afford **153**. Subsequently, the substitution of the halide by pyridine **1** provides the intermediate **154** which undergoes C–H activation followed by reductive elimination to furnish the C3-arylated product **152**.

### Heteroarylation

#### C-2 Heteroarylation

**Scheme 30:**
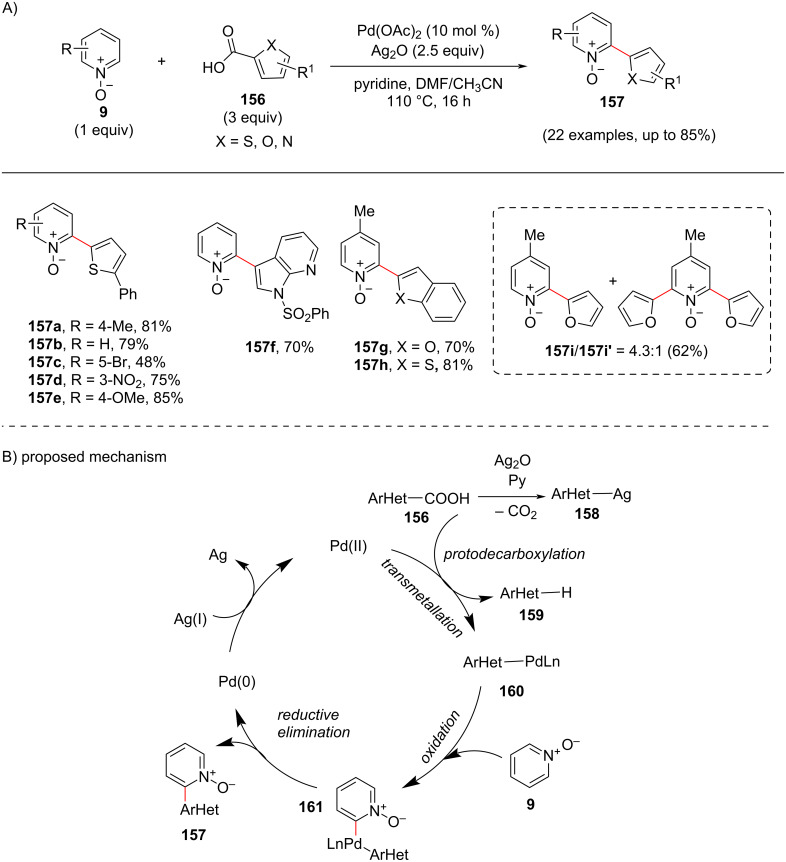
Pd-catalyzed pyridine *N*-oxide C–H arylation with heteroarylcarboxylic acids.

Heteroaryl groups are a common core in natural products and pharmaceuticals. In addition, the heterodiaryl systems widely occur in biologically important organic molecules, dyes, fragrances, advanced materials, and agrochemicals as well. Thus, the functionalization of the pyridine core with a heterocycle is a desirable transformation in organic synthesis. Manickam and co-workers [100] carried out a palladium-catalyzed decarboxylative *ortho*-(hetero)arylation of pyridine *N*-oxides **9** with heteroarylcarboxylic acids **156** (Scheme 30). The reaction showed good compatibility with various functional groups. The proposed mechanism (Scheme 30b) involves the silver-catalyzed decarboxylation of heteroaryl acid **156** followed by transmetalation providing palladium intermediate **160**. Further, C–H activation of pyridine *N*-oxide **9** provides intermediate **161** which upon reductive elimination furnishes the desired product **157** and regeneration of Pd(0) (Scheme 30b).

**Scheme 31:**
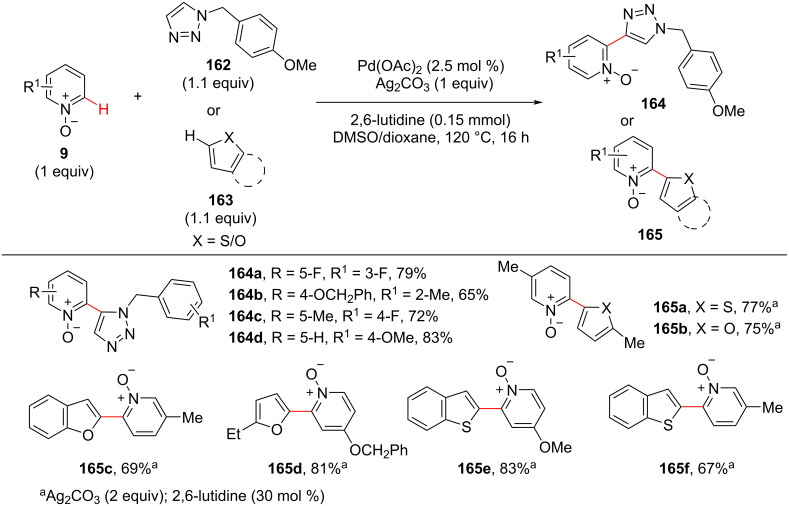
Pd-catalyzed C–H cross-coupling of pyridine *N*-oxides with five-membered heterocycles.

Later in 2014, Kuang and co-workers [101] developed a highly efficient and regioselective oxidative cross-coupling of pyridine *N*-oxides **9** with five-membered heterocycles **162** and **163** through a two-fold C–H activation under palladium catalysis. Silver carbonate and 2,6-lutidine were found to be an effective base and ligand, respectively, for providing the desired products **164** and **165** in good yields (Scheme 31).

**Scheme 32:**
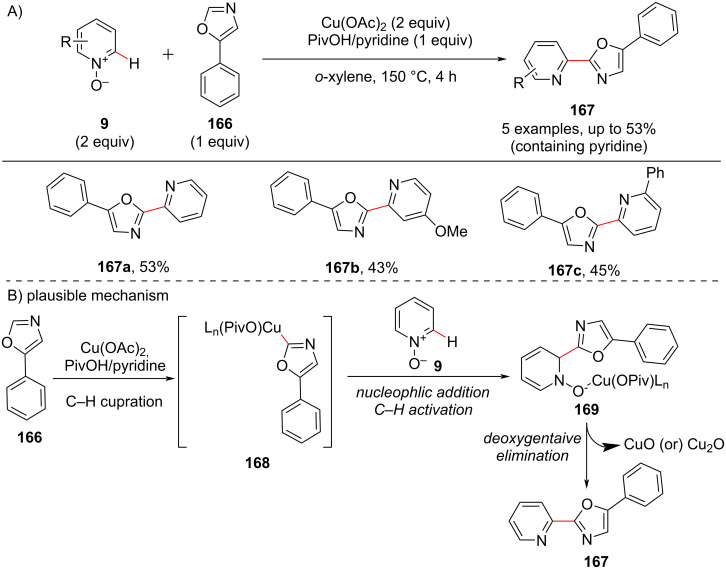
Cu-catalyzed dehydrative biaryl coupling of azine(pyridine) *N*-oxides and oxazoles.

In 2015, an economic route for copper-catalyzed biaryl coupling of azine(pyridine)-*N*-oxides **9** with oxazoles **166** was reported by Miura and group [102]. Although their work majorly covered quinoline *N*-oxide substrates, they also investigated three pyridine substrates in the reaction leading to the corresponding products in moderate yields (Scheme 32). The *N*-oxide plays a role as an activator and is subsequently eliminated via deoxygenative elimination furnishing the C-2-functionalized pyridines **167**. The reaction mechanism (Scheme 32b) involves the initial C–H-cupration of **166** producing an oxazolyl–copper intermediate **168**. Nucleophilic addition followed by C–H activation of **9** provides the hydroxy copper species **169**, which on deoxygenative elimination furnishes the desired product **167**.

#### C-3 Heteroarylation

**Scheme 33:**
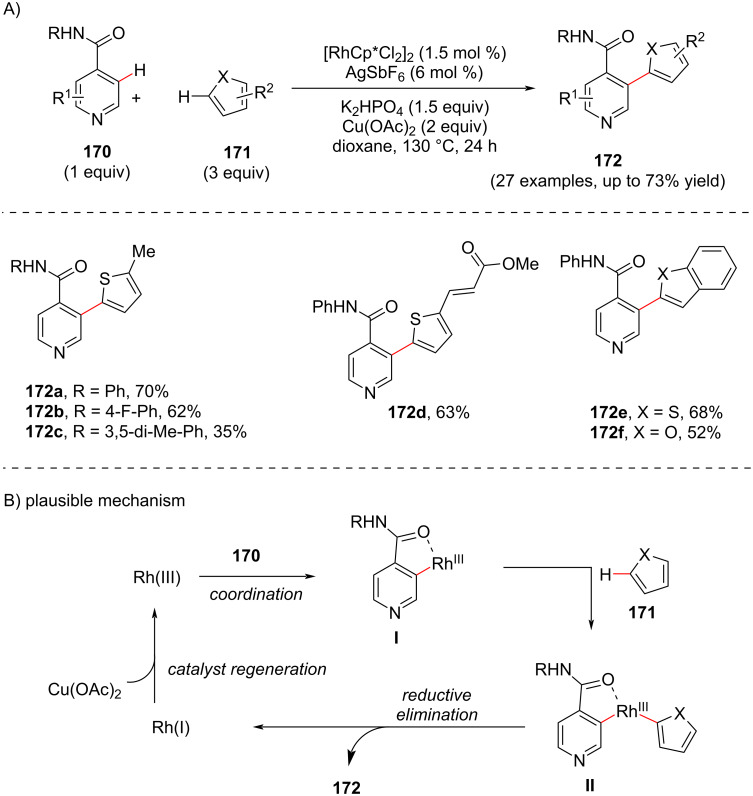
Rh(III)-catalyzed cross dehydrogenative C3-heteroarylation of pyridines.

In 2013, Su and co-workers [103] developed a catalytic methodology for the distal heteroarylation of pyridines **170** via Rh(III)-catalyzed dehydrogenative cross-coupling showcasing a good substrate scope (Scheme 33). Initially, their investigation involved evaluating the reaction between *N*-phenylisonicotinamide **170** and 2-methylthiophene **171** which resulted in the desired product **172**. The plausible mechanism (Scheme 33b) starts with the initial coordination of the pyridine directing group **170** with rhodium providing a five-membered rhodacyclic intermediate **I** which further forms the aryl–rhodium(III) complex **II** by reaction with **171**. Subsequently, this intermediate undergoes reductive elimination from the rhodium(III) center to furnish the desired *ortho*-C–H-arylated product **172** releasing a Rh(I) species. The Rh(III) species is regenerated in the presence of the copper salt.

**Scheme 34:**
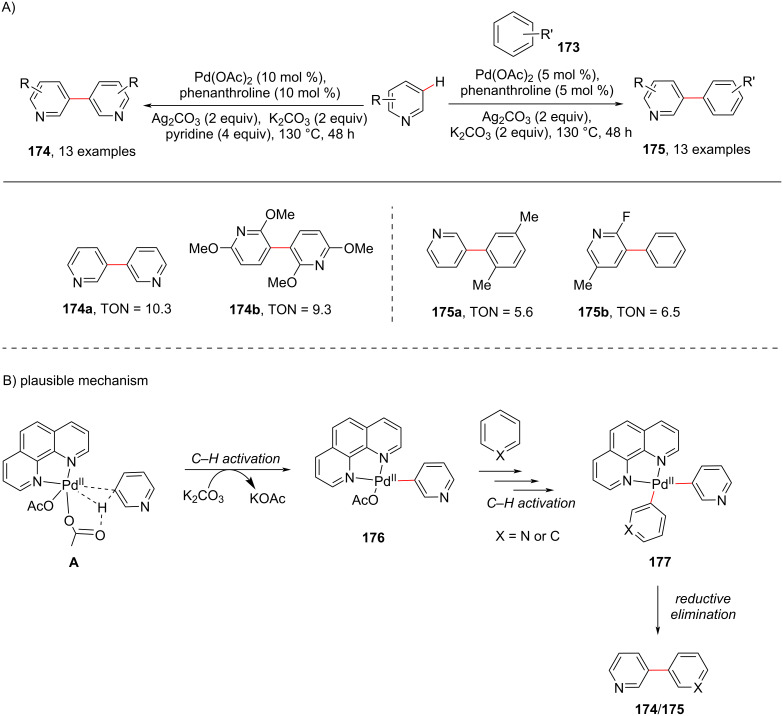
Pd-catalyzed C3-selective arylation of pyridines.

In another case of C3-(hetero)arylation, Yu and group [104] using palladium for C–H activation of pyridine with phenanthroline as a ligand developed a method in 2016 (Scheme 34). The authors achieved both arylation and heteroarylation at the C-3-position in pyridine and showcased the formation of bipyridines **174**. The mechanism is depicted in Scheme 34b, where the complex **A** undergoes C3–H activation to provide intermediate **176** which similarly undergoes one more step of C–H activation to provide the bi(hetero)arene–Pd(II) species **177** which undergoes reductive elimination furnishing the desired products **174**/**175**.

### C–H Annulation of pyridine to fused heterocycles

**Scheme 35:**
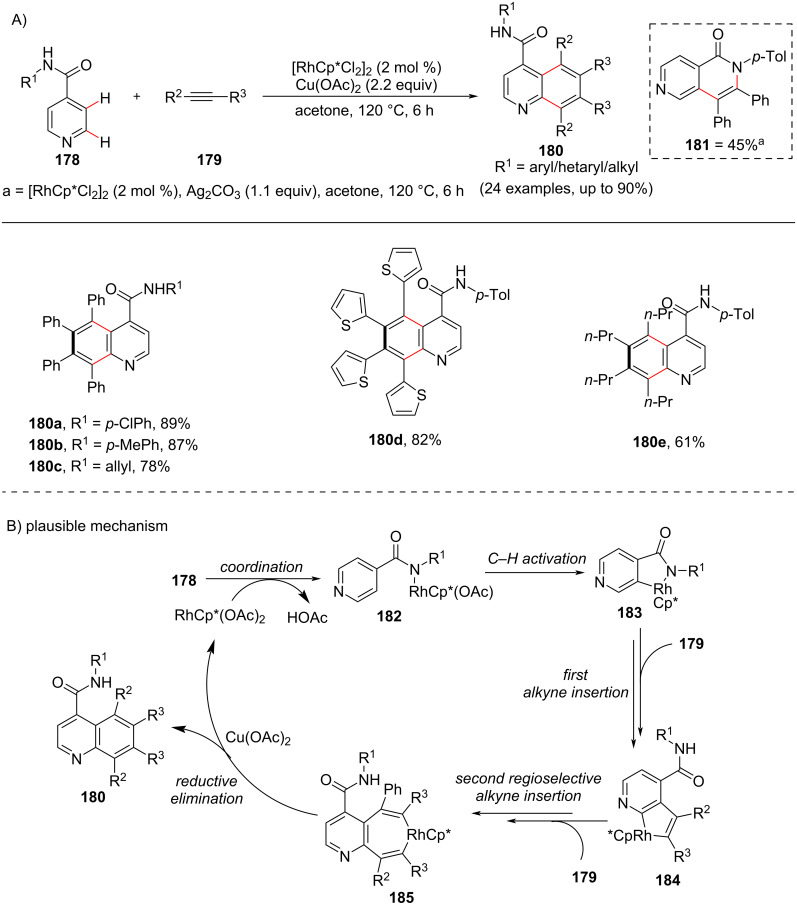
Rhodium-catalyzed oxidative C–H annulation of pyridines to quinolines.

Annulation reactions in organic synthesis have achieved great attention toward the construction of various carbocycles and heterocycles. These annulations can be either intermolecular or intramolecular and various substrates have been studied resulting in diverse products. Pyridine has been also reported for the construction of pyridine-fused heterocycles via C(sp^2^)–H functionalization and further annulation. In this aspect, considering the use of pyridines for the formation of quinolines and isoquinolines, an oxidant-dependent rhodium-catalyzed C–H annulation of pyridines with alkynes was reported by Li and co-workers [105] in 2011 for the direct synthesis of quinolines **180** and isoquinolines **181** involving a two-fold C–H activation of pyridine at the C2 and C3 position (Scheme 35a). Further, during optimization when silver additives like Ag_2_CO_3_, Ag_2_O, and AgOAc were used the reaction resulted in the formation of isoquinoline derivative **181**. In addition, the reaction showed high regioselectivity in the presence of unsymmetrical alkynes **179**. Different directing groups **178** were employed resulting in diversified products **180**. The proposed mechanism (Scheme 35b) involves coordination of rhodium with isonicotinamide **178** and subsequent *ortho*-C–H activation generating the five-membered rhodacycle **183**. Next, first alkyne **179** insertion results in the five-membered rhodacycle **184** which is followed by a second regioselective insertion of alkyne **179** into the Rh–C bond of **184** providing the seven-membered cyclic intermediate **185**. Further reductive elimination furnishes the quinoline product **180** and a Rh(I) species, with the latter being oxidized by Cu(II) to complete the catalytic cycle.

**Scheme 36:**
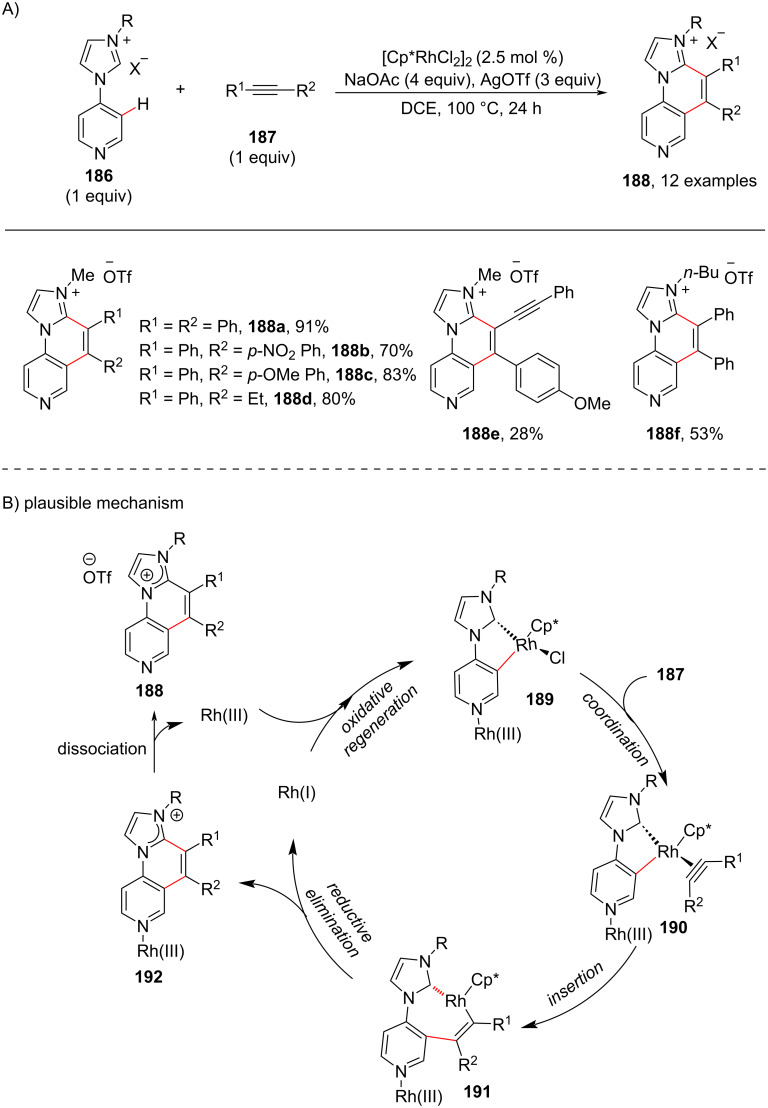
Rhodium-catalyzed and NHC-directed C–H annulation of pyridine.

Next, considering the role of N-heterocyclic carbene (NHC) ligands acting as directing group as well as functionalizing unit in arene C–H functionalization reactions with alkynes, Choudhury and group [106] in 2015 developed a protocol for the intermolecular C–H annulation of NHC-substituted pyridines with a variety of internal alkynes **187** under rhodium catalysis for the synthesis of annulated and highly decorated pyridines **188** (Scheme 36). The authors used the N-heterocyclic carbene ligand as directing group to prepare imidazo[1,2-*a*][1,6]naphthyridine motifs **188** as desired products. Based on the experimental results and annulation chemistry a catalytic mechanism has been proposed (Scheme 36b) that involves the C3 hydrogen of pyridine undergoing a cyclorhodation with the catalyst in the presence of NaOAc, directed by in-built NHC ligand coordination to provide intermediate **189**. The further coordination of **189** with the alkyne **187** results in intermediate **190** and subsequent insertion provides rhodacycle intermediate **191** which undergoes reductive elimination to furnish the product **188** via dissociation of intermediate **192** along with oxidative regeneration of **189** (Scheme 36b).

**Scheme 37:**
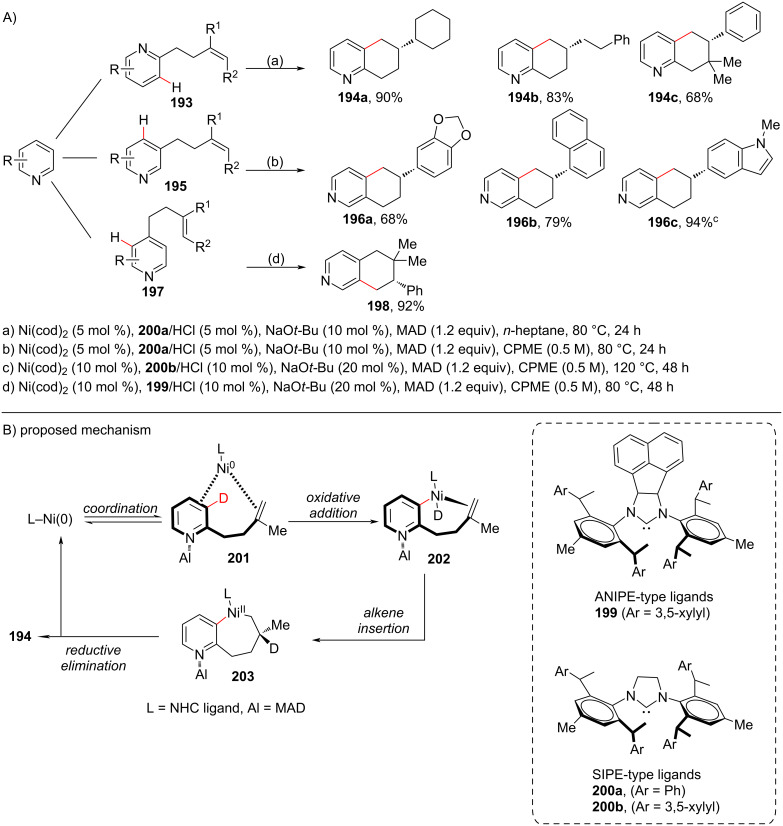
Ni/NHC-catalyzed regio- and enantioselective C–H cyclization of pyridines.

In 2019, using NHC ligands, a protocol for the regio- and enantioselective C–H cyclization of pyridines was reported by Shi and co-workers [107] toward the direct asymmetric pyridine C–H alkylation (Scheme 37). The authors found that alkene-tethered C2 pyridine **193**, C3 pyridine **195** and C4 pyridine **197** can undergo *endo*-cyclization reactions in the presence of Ni(cod)_2_, a chiral NHC ligand, and MAD as Lewis acid to afford optically active 5,6,7,8-tetrahydroquinolines **194** and 5,6,7,8-tetrahydroisoquinolines **196** and **198**. The *endo*-selective annulation approach was compatible with various tethered alkenes, such as 1,1-disubstituted alkenes, styrene, diene, trisubstituted alkene and enamines. To get insights into the mechanism the authors conducted additional experiments including deuterium labelling reactions and proposed the mechanism depicted in Scheme 37b. Initially, the sterically bulky additive MAD coordinates to the pyridine nitrogen, which pushes the tethered alkene close to the nickel center subsequently providing the intermediate **201**. Then, the C–D bond on cleavage via oxidative addition of Ni(0) forms the Ni–D species **202** which after anti-Markovnikov hydronickelation of the alkene provides the seven-membered cyclic intermediate **203**. Subsequent reductive elimination furnishes the endo-annulated product **194** (Scheme 37b).

**Scheme 38:**
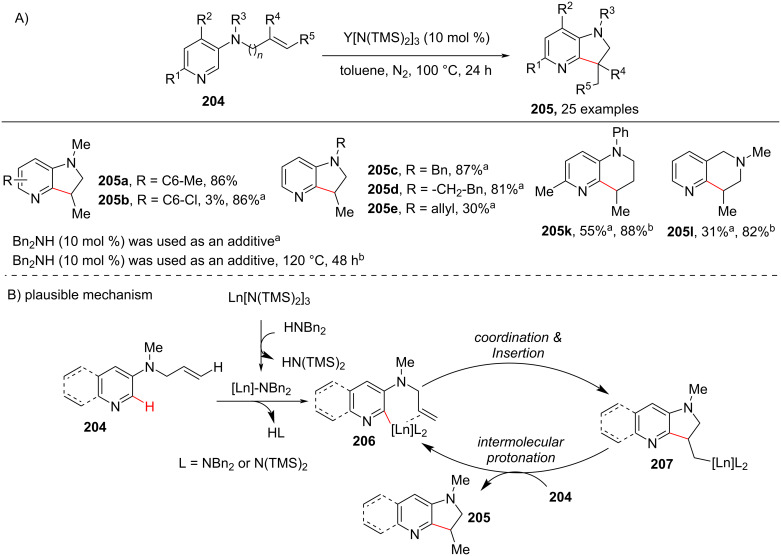
Rare earth metal-catalyzed intramolecular C–H cyclization of pyridine to azaindolines.

Out of various pyridine-fused heterocyclic hybrids, azaindolines are important scaffolds in natural products and pharmaceuticals serving different biological activities. Hence, looking at the importance of azaindolines in drug discovery a protocol of rare earth metal-catalyzed intramolecular insertion of the pyridine C–H bond into unactivated vinyl C–H bonds has been developed by Chen and co-workers [108] (Scheme 38). Using this protocol azaindolines **205** were accessed in moderate to excellent yields and also naphthyridine derivatives (**205k** and **205l**) were synthesized. In the proposed mechanism, the initial deprotonation of HNBn_2_ by Ln[N(TMS)_2_]_3_ provided the lanthanide amide. Activation of the vinyl-substituted pyridin-3-amine **204** by the lanthanide amide gives a lanthanide–pyridine complex **206**. Then, coordination and sequential insertion of C=C into the Ln–pyridine bond of **206** provided intermediate **207**, which undergoes intermolecular protonation with **204** to afford the desired product **205** and regenerating the lanthanide species (Scheme 38b).

#### C(sp^2^)–H Functionalization of pyridine rings in bipyridine systems

**Scheme 39:**
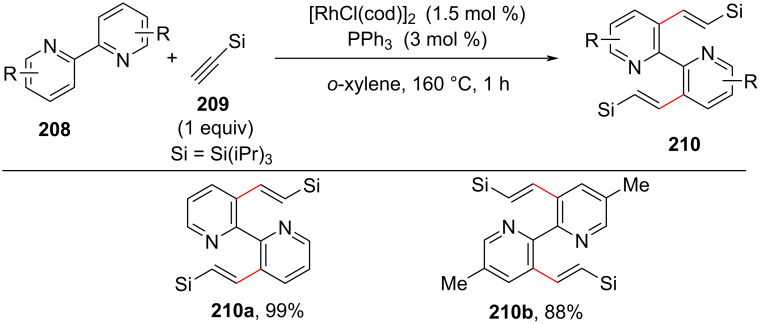
Rh-catalyzed alkenylation of bipyridine with terminal silylacetylenes.

The functionalization and synthesis of bipyridine derivatives is of great interest and importance in synthetic chemistry. These compounds are well-studied for their roles as chelating ligands in transition-metal-catalyzed reactions, coordination chemistry including materials science [109–110]. The challenge associated with the C–H functionalization of bidentate molecules is the finding strategy in the subduing the high activation barrier of rollover cyclometallation pathway. In this section we discuss the C(sp^2^)–H functionalization of the pyridine ring in bipyridine ring systems. In early 2009 Miura and co-workers [111] reported the rhodium-catalyzed regioselective reaction of aryl-*N*-heterocycles and aromatic imines with terminal silylacetylenes **209** to synthesize C–H-alkenylated products **210**. The terminal silylacetylenes were employed as effective substrates for catalytic cross-dimerization reactions. The reaction was performed in the presence of [RhCl(cod)]_2_ (3 mol %), taking PPh_3_ or (4-ClC_3_H_4_)_3_P as the ligand at 160 °C, for about 48 h (Scheme 39). This work provides an effective way for preparing C–H-alkenylated bipyridines **210**.

**Scheme 40:**
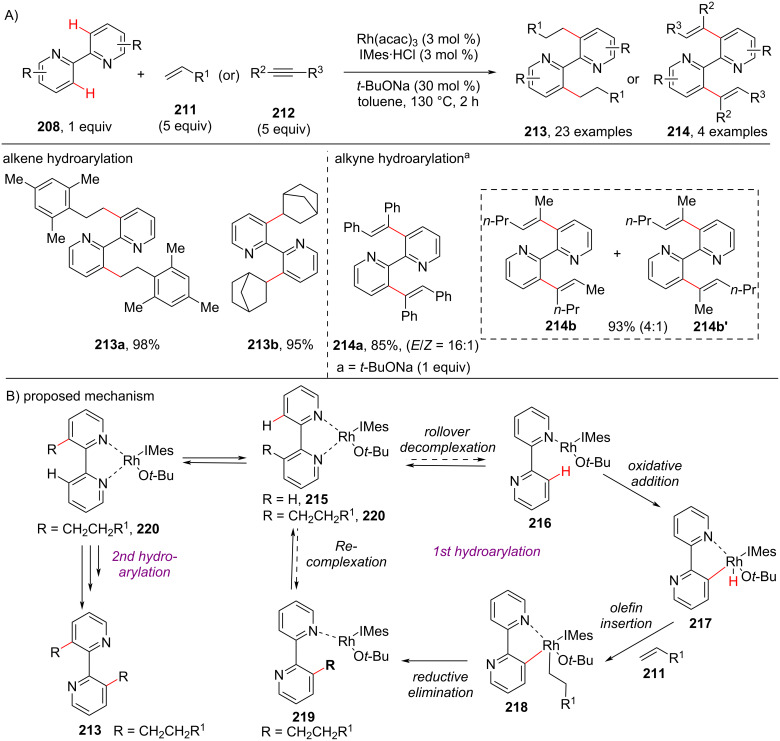
Rollover cyclometallation in Rh-catalyzed pyridine C–H functionalization.

In 2012, a hydroarylation of alkenes **211** and alkynes **212** with 2,2′-bipyridines **208** and 2,2′-biquinolines was reported by Chang and co-workers [112] in the presence of Rh(acac)_3_ as catalyst, IMes·HCl (3 mol %) as ligand and *t*-BuONa (30 mol %) in toluene for 2 h (Scheme 40). The authors demonstrated theoretically and mechanistically the important role of the NHC ligand in the resultant catalyst Rh(NHC) for the hydroarylation of alkenes and alkynes with chelating 2,2-bipyridine and 2,2-biquinoline molecules. The experimental studies revealed that the *trans*-effect of the NHC ligand in the complex assisting in the reduced energy barrier of a rollover cyclometallation pathway and results in selective and efficient hydroarylation of the alkenes and alkynes. This was the first report for the role of a “rollover” cyclometallation pathway catalytically leading to double C–H bond functionalization of chelating molecules under action of a Rh(NHC) system. Based on the computational studies and experimental data, the proposed mechanism (Scheme 40b) describes that the Rh(I) complex ligated to *tert*-butoxide and NHC (IMes·HCl) **215** is a catalytically active species. The Rh–hydride species **217** is formed after oxidative addition via C–H-bond cleavage followed by olefin insertion to form intermediate **218**, which on subsequent reductive elimination results in the formation of monoalkylated bipyridine **219**. This intermediate reenters into another cycle of hydroarylation by starting as bidentate complex **220** and finally furnishing the desired bishydroarylated product **213** (Scheme 40b).

**Scheme 41:**
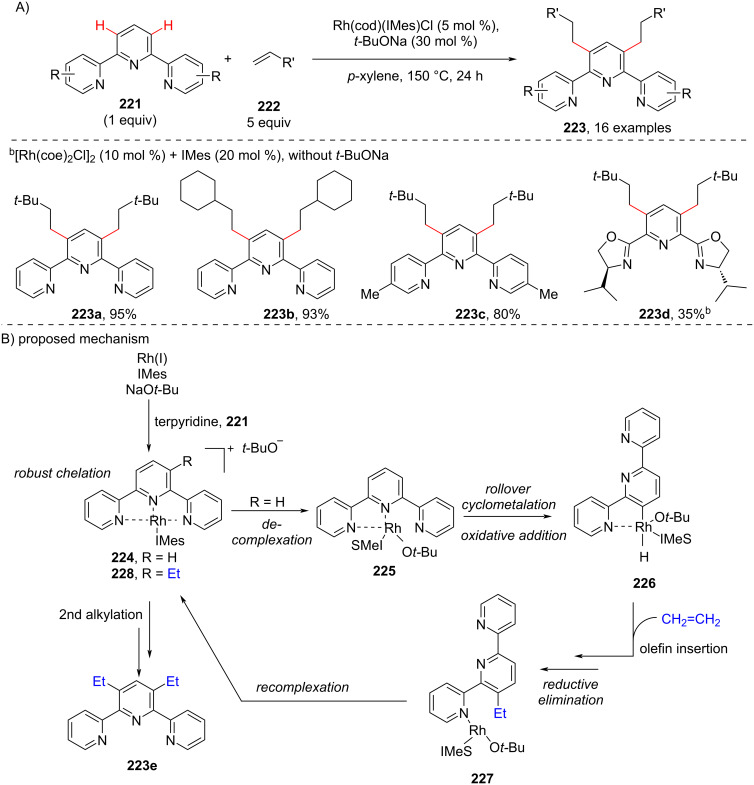
Rollover pathway in Rh-catalyzed C–H functionalization of *N*,*N*,*N*-tridentate chelating compounds.

Next, a protocol for the selective and catalytic C–H functionalization of *N*,*N*,*N*-tridentate chelating compounds using a rollover cyclometallation strategy was reported by the same group in 2016 [113]. The reaction involves the Rh-catalyzed alkylation of 2,2’:6’,2”-terpyridine **221** with 3,3-dimethyl-1-butene coupled in the presence of a catalytic amount of *t*-BuONa providing the mono- and dialkylated products in low combined yields. The alkylation of terpyridines with aliphatic olefins **222** afforded only anti-Markonikov linear products **223** (Scheme 41). The authors also expanded their study to tridentate heteroarenes. Delightfully, they observed the dialkylated products **223** in good yields. The plausible reaction mechanism (Scheme 41b) was explained by the formation of a cationic Rh–terpyridine complex **224** generated from terpyridine **221** and a Rh(NHC) species formed from the Rh(I) precursor and the NHC in the presence of an external base and successive decomplexation of **224** provides complex **225**. The latter undergoes an initial key rollover cyclometallation followed by oxidative addition leading to the metal–hydride intermediate **226** which on olefin insertion and subsequent reductive elimination resulted in the monoalkylated rhoda complex **227**. Complex **227** then undergoes recomplexation to form **228** and enters the subsequent catalytic cycle furnishing the bisalkylated product **223**.

**Scheme 42:**
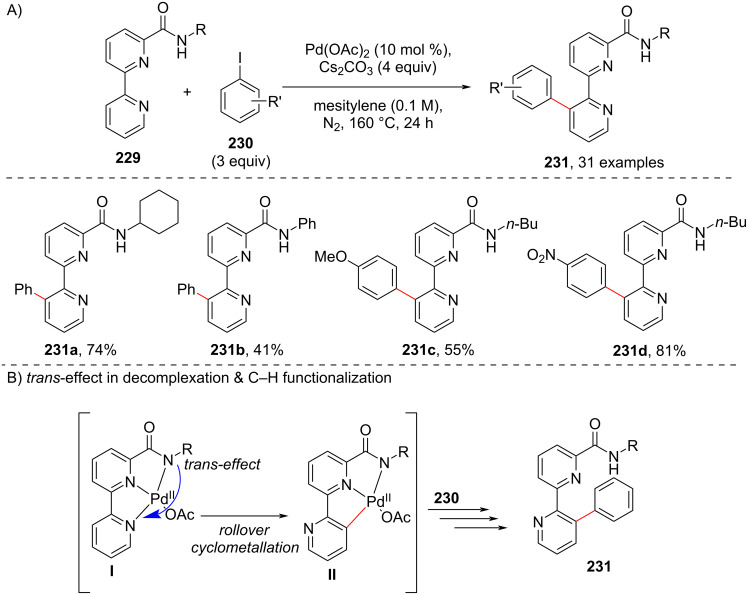
Pd-catalyzed rollover pathway in bipyridine-6-carboxamides C–H arylation.

In 2018, Cheng and co-workers [114] reported a straight forward approach to 3’-aryl-2,2’-bipyridine-6-carboxamide derivatives **231** with exclusive selectivity starting from 2,2’-bipyridine-6-carboxamides **229** under Pd catalysis (Scheme 42). The arylation reaction of *N*-butyl-2,2’-bipyridine-6-carboxamide with iodobenzene **230** in the presence of Pd(OAc)_2_ as catalyst, Cs_2_CO_3_ as a base in DMSO at 160 °C furnished the desired products **231** (Scheme 42). It was found that non-polar solvents resulted in good yields of the products **231**. It is reported that 2,2’-bipyridine-6-carboxamides **229** can bind to the transition metal, such as Pd(II), to form stable *N*,*N*,*N*-chelates **I** (Scheme 42b). The amide moiety of the *N*,*N*,*N*-chelates **I** exerts a strong *trans*-effect which weakens the Pd(II)–pyridyl bond trans to the amide anion, thus, allowing the decomplexation to afford complex **II** which is key intermediate for furnishing the desired C–H functionalization product (Scheme 42b).

**Scheme 43:**
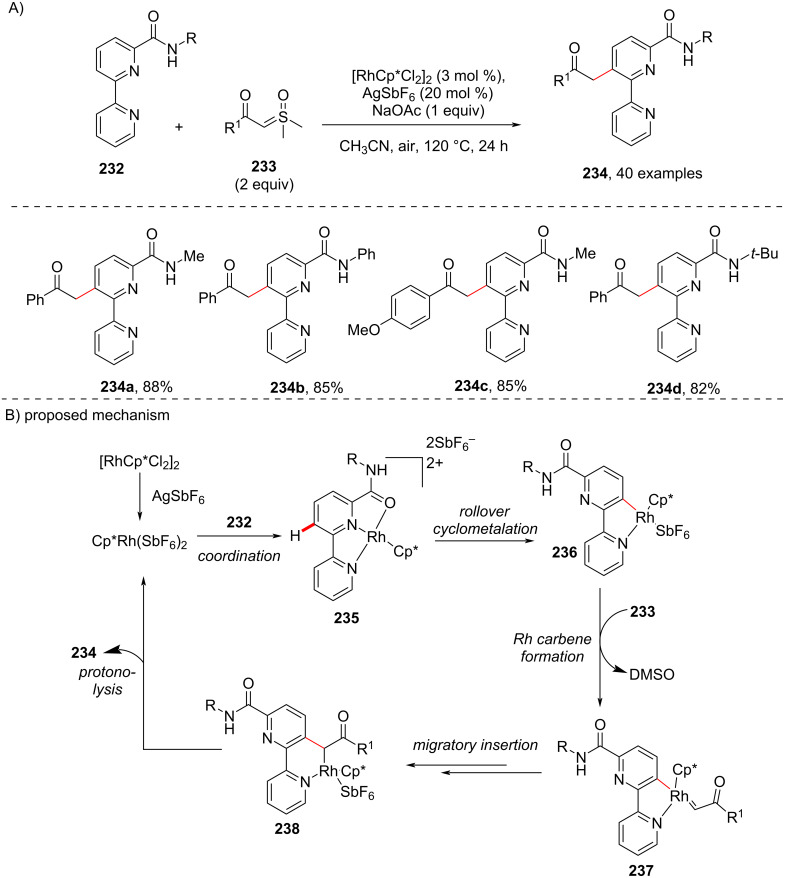
Rh-catalyzed C3-acylmethylation of bipyridine-6-carboxamides with sulfoxonium ylides.

In 2019, Cheng and co-workers reported an approach for the C3-selective acylmethylation of [2,2”-bipyridine]-6-carboxamides **232** with sulfoxonium ylides **233** in the presence of a Rh(III) catalyst (Scheme 43) [115]. Sterically hindered amide directing groups were also well tolerated under the optimal conditions. A H/D exchange reaction exclusively at the C3-position suggested C–H-bond cleavage is reversible. The catalytic cycle involves the coordination of the carboxamide **232** with the Rh(III) species affording Rh(III) complex **235**, which on rollover cyclometalation gives the complex **236**. The addition of sulfoxonium ylide **233** to the intermediate complex **236** generates the Rh–carbene complex **237** with the release of DMSO and further migratory insertion of complex **237** and subsequent protonolysis furnishes the acylmethylated product **234** (Scheme 43b).

**Scheme 44:**
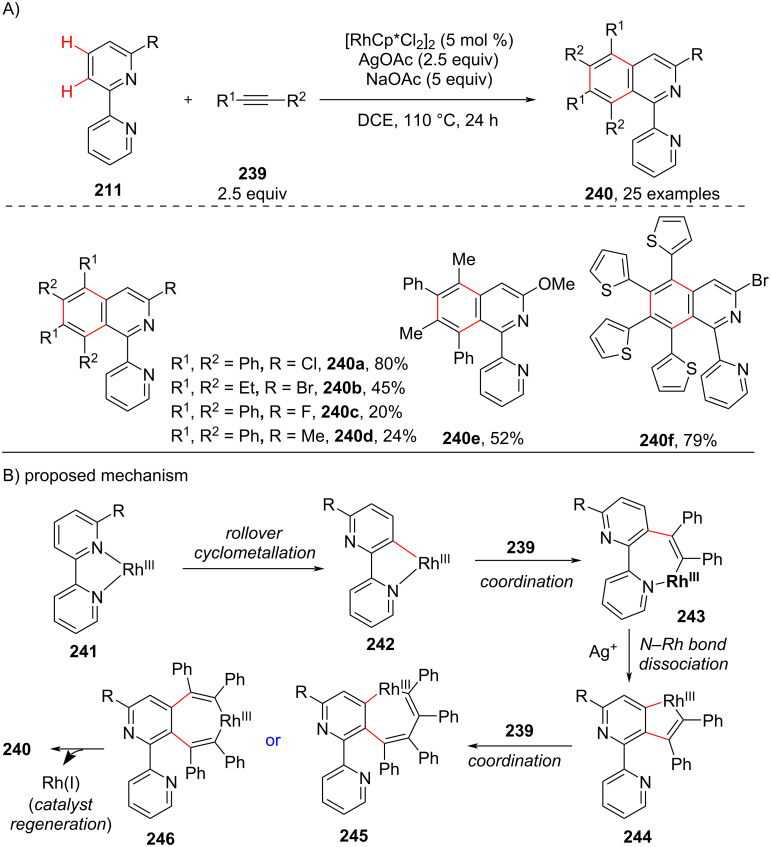
Rh-catalyzed C–H functionalization of bipyridines with alkynes.

Recently, in 2020, Zhu and co-workers [116], developed a novel annulation reaction of bipyridine systems **211** with alkynes **239** via a Rh(III)-catalyzed dual C–H functionalization. The authors have initiated their studies with 6-bromo-2,2’-bipyridine as their model substrate and with diphenylacetylene as coupling partner. The optimized conditions included [RhCp*Cl_2_]_2_ (5 mol %), AgOAc (2.5 equiv), NaOAc (5 equiv) in DCE, at 110 °C for 24 h to obtain the annulated product **240** (Scheme 44). The proposed mechanism (Scheme 44b) involves the formation of Rh(III) complex **241** by coordination of the bipyridine with rhodium and complex **241** via a rollover cyclometallation process gives the intermediate **242**. It was suggested that the substitution at the 6 position of the bipyridine ring system facilitates the rollover cyclometallation process by weakening the Rh–N bond. Next, intermediate **242** coordinates with alkyne **239** to give the seven-membered rhodacycle **243**. The excess Ag^+^ help in the dissociation of the N–Rh bond in complex **243** and give the five-membered rhodacyclic intermediate **244** which again coordinates with the alkyne **239** furnishing another seven-membered rhodacyclic intermediate **245** or **246**. Finally, reductive elimination delivers the desired product **240**.

**Scheme 45:**
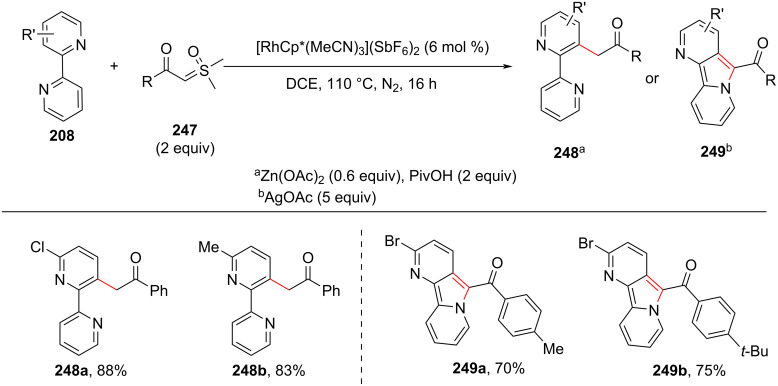
Rh-catalyzed C–H acylmethylation and annulation of bipyridine with sulfoxonium ylides.

In the subsequent year, the same group reported a method for the rhodium-catalyzed acylmethylation of bipyridines [117]. The group has demonstrated a switchable reaction, wherein changing the additive can deliver the acylmethylated product **248** or the annulation product pyrido[2,3-*a*]indolizine **249** (Scheme 45). Under action of the Rh(III) catalyst, zinc acetate and PivOH as additives, the acylmethylation of bipyridines takes place at the C-2 position to furnish acylmethylated products **248** and the reaction was found suitable for various substrates. On the other hand, the usage of silver acetate as an additive provided the annulated (intramolecular cyclization of bipyridine) product **249**.

### Miscellaneous reactions

#### C–H Borylation

**Scheme 46:**
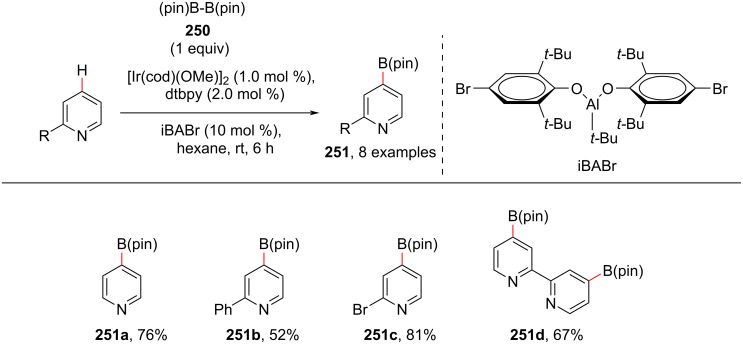
Iridium-catalyzed C4-borylation of pyridines.

Due to the broad utilities of arylboronic esters in organic synthesis, various protocols have been reported till date for their incorporation into an organic molecule. In 2017, Nakao and group reported a method for the iridium-catalyzed *para*-C–H borylation of pyridines using bis(pinacolato)diboron (**250**) for the synthesis of borylated pyridines **251**, which are important intermediates for various derivatization reactions (Scheme 46) [118]. In common, site-selective borylations have been in less focus, due to the lack of suitable strategies, however, this group achieved the *para*-selective borylation of pyridines using a cooperative catalyst strategy. The authors used [Ir(cod)(OMe)]_2_ as a metal catalyst, along with a sterically bulky Lewis acid such as methylaluminum bis(2,6-di-*tert*-butyl-4-methylphenoxide) as a cooperative catalyst.

**Scheme 47:**
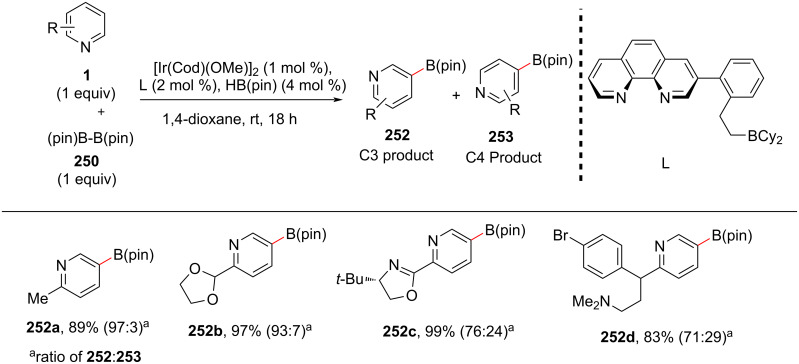
C3-Borylation of pyridines.

Later, in 2019, the same group reported a protocol for the selective C5(C3)-borylation of pyridines under iridium–Lewis acid bifunctional catalysis (Scheme 47) [119]. With the optimized conditions in hands, the authors screened for the substrate scope of substituted pyridines. Also, they employed the reported protocol for the late-stage functionalization of brompheniramine (**252d**), an antihistaminic drug.

#### C–H Silaboration

**Scheme 48:**
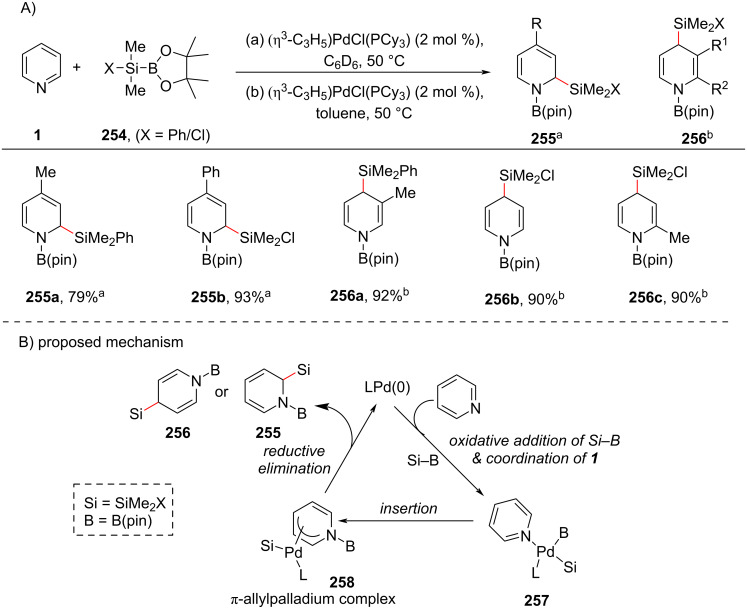
Pd-catalyzed regioselective synthesis of silylated dihydropyridines.

In 2011, a protocol for the synthesis of highly functionalized dihydropyridines via palladium-catalyzed silaboration providing silylated dihydropyridines **255** and **256** (Scheme 48) was developed by Suginome and co-workers [120]. This reaction involved a dearomatizing conversion of pyridines to dihydropyridines under mild conditions with the introduction of a silyl group on a carbon atom of pyridine ring. Various pyridines were subjected to this silaboration using the Pd/PCy_3_ catalytic system providing the corresponding products in good yields. The proposed mechanism (Scheme 48b) involves the oxidative addition of silylboronic ester **254** to Pd(0) and coordination of pyridine **1** providing the intermediate **257** which on further regioselective insertion of pyridine into the Pd–B bond resulted in the π-allyl palladium complex **258**. Subsequent reductive elimination furnishes the silaboration products **255** and **256** with the regeneration of Pd(0).

## Conclusion

Significant progress has been made in the area of *ortho*- and distal C–H-functionalization of pyridines, as evidenced by the reactions outlined in this review. The previous research and their mechanistic insight provided us with more information to approach the new avenue of catalytic C−H functionalization of the pyridine nucleus. The challenges still remain for the distal C–H functionalization, particularly at the C4 position. Even the directing group on pyridine ring system has been less explored for *ortho*- or distal C–H functionalization. Although the C–H functionalization with transition-metal catalysis and rare earth metal catalysis has advanced, the functionalization of the pyridine ring system can further be explored by employing new catalytic systems and merging of different strategies. Taking this into account, we hope that the efforts for the development of novel protocols for the preparation and incorporation of functionalized pyridine scaffolds will continue and could be applicable for applications in industry.
